# Circular RNA Gtdc1 Protects Against Offspring Osteoarthritis Induced by Prenatal Prednisone Exposure by Regulating SRSF1‐Fn1 Signaling

**DOI:** 10.1002/advs.202307442

**Published:** 2024-03-22

**Authors:** Liang Liu, Yuntian Hong, Chi Ma, Fan Zhang, Qingxian Li, Bin Li, Hangyuan He, Jiayong Zhu, Hui Wang, Liaobin Chen

**Affiliations:** ^1^ Department of Orthopedic Surgery Joint Disease Research Center of Wuhan University Zhongnan Hospital of Wuhan University Wuhan 430071 China; ^2^ Department of Gastroenterology Zhongnan Hospital of Wuhan University Wuhan 430071 China; ^3^ Hubei Provincial Key Laboratory of Developmentally Originated Disease Wuhan 430071 China; ^4^ Department of Pharmacology Wuhan University School of Basic Medical Sciences Wuhan 430071 China

**Keywords:** alternative RNA splicing, chondrodysplasia, circular RNA, osteoarthritis, prenatal prednisone exposure

## Abstract

Chondrodysplasia is closely associated with low birth weight and increased susceptibility to osteoarthritis in adulthood. Prenatal prednisone exposure (PPE) can cause low birth weight; however, its effect on offspring cartilage development remains unexplored. Herein, rats are administered clinical doses of prednisone intragastrically on gestational days (GDs) 0–20 and underwent long‐distance running during postnatal weeks (PWs) 24–28. Knee cartilage is assayed for quality and related index changes on GD20, PW12, and PW28. In vitro experiments are performed to elucidate the mechanism. PPE decreased cartilage proliferation and matrix synthesis, causing offspring chondrodysplasia. Following long‐distance running, the PPE group exhibited more typical osteoarthritis‐like changes. Molecular analysis revealed that PPE caused cartilage circRNomics imbalance in which circGtdc1 decreased most significantly and persisted postnatally. Mechanistically, prednisolone reduced circGtdc1 expression and binding with Srsf1 to promote degradation of Srsf1 via K48‐linked polyubiquitination. This further inhibited the formation of EDA/B^+^Fn1 and activation of PI3K/AKT and TGFβ pathways, reducing chondrocyte proliferation and matrix synthesis. Finally, intra‐articular injection of offspring with AAV‐circGtdc1 ameliorated PPE‐induced chondrodysplasia, but this effect is reversed by Srsf1 knockout. Altogether, this study confirms that PPE causes chondrodysplasia and susceptibility to osteoarthritis by altering the circGtdc1‐Srsf1‐Fn1 axis; in vivo, overexpression of circGtdc1 can represent an effective intervention target for ameliorating PPE‐induced chondrodysplasia.

## Graphical abstract

Prenatal prednisone exposure can dose‐dependently reduce chondrocyte proliferation and extracellular matrix synthesis, leading to chondrodysplasia and susceptibility to OA. In mechanism, the alteration of circGtdc1‐Srsf1‐EDA/B^+^Fn1 axis, mediated by prednisolone, contributed to the inhibition of chondrocyte proliferation and matrix synthesis, causing chondrodysplasia and an increased susceptibility to OA. CircGtdc1 can represent an effective intervention target for ameliorating PPE‐induced chondrodysplasia.

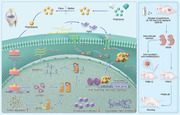

## 1 Introduction

Osteoarthritis (OA) is the most common chronic joint disorder characterized by cartilage and subchondral bone destruction, which occurs commonly in females.^[^ ^1^ ^]^ As of 2015, ≈237 million people worldwide suffered from OA, representing 3.3% of the global population.^[^ ^2^ ^]^ However, owing to insufficient research on its etiology and pathogenesis, effective strategies for the early treatment of OA are lacking.^[^ ^1^ ^]^ Articular cartilage primarily develops during embryogenesis and gradually decreases in thickness and chondrocyte abundance after birth.^[^ ^3^ ^]^ Moreover, the self‐repair ability of the articular cartilage is significantly diminished following injury due to poor vascularization. Accordingly, cartilage development during pregnancy critically impacts adult cartilage quality, potentially leading to OA development.^[^ ^4^ ^]^ Increasing epidemiological evidence suggests that OA in adults has an intrauterine origin. That is, individuals with low birth weight have a significantly increased incidence of OA of the hands, lumbar spine, and hip joints in adulthood, as well as noticeable knee osteophyte formation.^[^ ^5^ ^]^ Our previous studies have found that prenatal exposure to various exogenous substances (caffeine, nicotine, ethanol, and dexamethasone) and dietary restrictions can lead to poor cartilage quality and susceptibility to OA in adult offspring,^[^ ^6^ ^]^ the mechanism by which involves programming changes of transforming growth factor beta (TGFβ) and insulin‐like growth factor 1 signaling pathways within fetal articular cartilage during the intrauterine period.^[^ ^6e,h^ ^]^ Therefore, OA is of fetal origin and may be associated with low birth weight and poor cartilage quality in offspring induced by adverse intrauterine environments.

Prednisone, a synthetic glucocorticoid, has multiple functions, including anti‐inflammatory, anti‐allergic, and inhibitory effects on connective tissue hyperplasia.^[^ ^7^ ^]^ Clinically, prednisone is widely used during pregnancy for the treatment of various conditions, such as immune anemia, asthma, systemic lupus erythematosus, antiphospholipid syndrome, and rheumatoid arthritis.^[^ ^8^ ^]^ However, the adverse outcomes associated with prenatal prednisone exposure (PPE) have recently received increasing attention.^[^ ^8d^ ^]^ To exert its biological effects, prednisone is metabolized to prednisolone in vivo by the hepatic 11β‐hydroxysteroid dehydrogenase type 1 (11β‐HSD1) enzyme.^[^ ^9^ ^]^ Prednisone and prednisolone can cross the placenta,^[^ ^10^ ^]^ causing adverse effects on fetal organ development.^[^ ^8^, ^9^ ^]^ Clinical and laboratory studies have shown that PPE can cause low birth weight in offspring,^[^ ^9^, ^11^ ^]^ which is significantly negatively correlated with its dose and unrelated to maternal diseases or dysfunction.^[^ ^9a^ ^]^ Furthermore, PPE can result in preterm birth,^[^ ^11^ ^]^ neonatal acute adrenal insufficiency,^[^ ^12^ ^]^ offspring bone marrow hypoplasia,^[^ ^13^ ^]^ suppression of immune system development,^[^ ^9^, ^13^ ^]^ genital and urethral dysplasia,^[^ ^14^ ^]^ cleft lip and palate.^[^ ^8^, ^9^ ^]^ However, the effects of PPE on cartilage development have not been reported. Our previous work showed that exposure to another synthetic corticosteroid, dexamethasone, during pregnancy, also caused low offspring birth weight, chondrodysplasia, and increased susceptibility to OA in adulthood.^[^ ^15^ ^]^ So, does PPE also cause chondrodysplasia in offspring and susceptibility to OA in adulthood?

Circular RNAs (circRNAs) are loop RNA molecules formed by covalently closing the 5ʹ and 3ʹ ends. This unique structure makes them highly stable and resistant to degradation by exonuclease RNase R.^[^ ^16^ ^]^ Since the first report on the biological function of circRNAs in 2013,^[^ ^17^ ^]^ multiple studies have revealed their crucial roles in the development of diverse tissues and organs, as well as in pathological changes related to diseases.^[^ ^18^ ^]^ For instance, circSamd4 can interact with the transcription factors purine‐rich binding protein alpha and beta to block their entry into the nucleus, promoting myocyte differentiation and regulating skeletal muscle development.^[^ ^19^ ^]^ Further, circHomer 1 can regulate synaptic structural changes, affecting the plasticity and development of the central nervous system.^[^ ^20^ ^]^ Meanwhile, circRNA422^[^ ^21^ ^]^ and circATRNL1^[^ ^22^ ^]^ can promote the osteogenic and chondrogenic differentiation of bone marrow mesenchymal stem cells. Additionally, circPDE4B promotes the interaction between RIC8 guanine nucleotide exchange factor A and midline 1 through molecular scaffolding, preventing degradation of the cartilage matrix and promoting repair.^[^ ^23^ ^]^ Moreover, circSERPINE2 regulates chondrocyte apoptosis and the balance between matrix synthesis and catabolism by targeting miR‐1271‐5p and the E26 transformation‐specific‐related gene, preventing OA development.^[^ ^24^ ^]^ Therefore, circRNAs can influence the development of multiple organs and may participate in OA development. However, the impact of circRNAs on cartilage development and their potential role in mediating susceptibility to adult‐onset OA remain unclear.

In this study, Wistar rats were administered different doses of prednisone by oral gavage during gestational days (GDs) 0–20, followed by a second strike (long‐distance running) during postnatal weeks (PWs) 24–28. Cartilage quality and OA susceptibility were assessed at GD20, PW12, and PW28. Meanwhile, using circRNA high‐throughput sequencing and in vitro and in vivo experiments, a circRNA that exhibited significant changes and elicited the greatest impact on the phenotype was screened and identified, and the underlying mechanisms by which this circRNA mediated PPE‐induced chondrodysplasia and susceptibility to OA in adulthood was investigated. Finally, an adeno‐associated virus that regulates the circRNA expression was injected into the rat joint cavity to assess therapeutic effects on fetal‐derived chondrodysplasia. The present study aimed to confirm the effects of PPE on cartilage development and OA susceptibility through in vivo and in vitro experiments and to explore the mechanism of epigenetic programming in utero, to provide an experimental and theoretical foundation for the early prevention and treatment of fetal‐derived OA.

## 2 Results

### 2.1 PPE Induces Poor Cartilage Qualities in Offspring Rats and Susceptibility to OA in Adulthood

**Figure 1:**
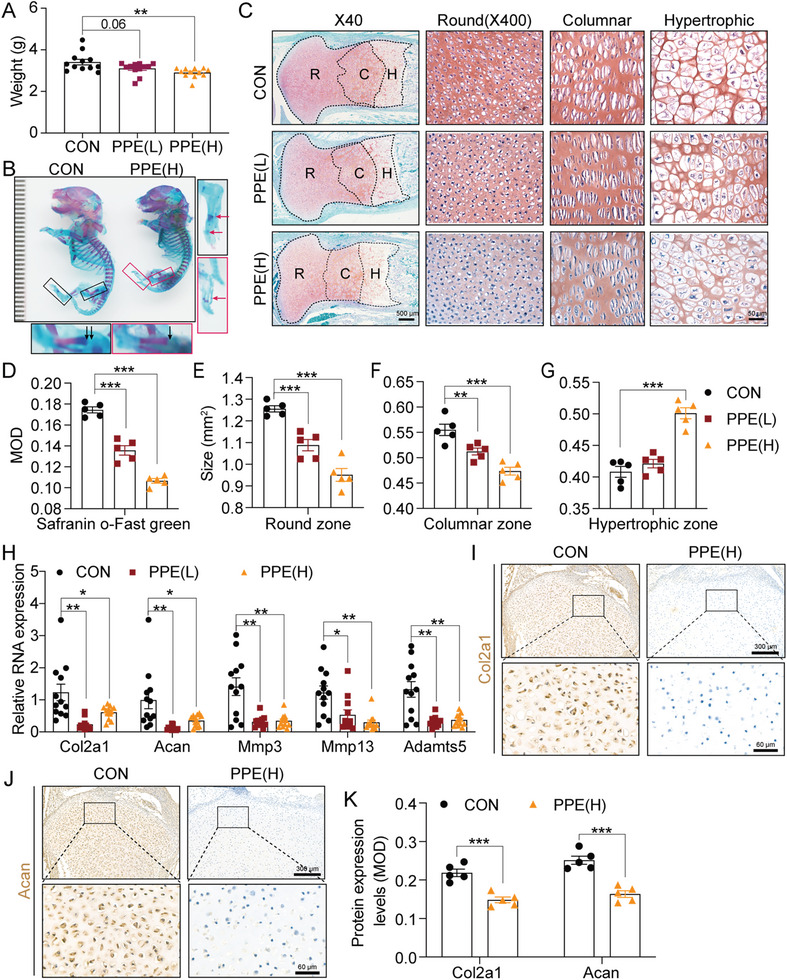
Effects of PPE on cartilage quality in fetal rats on GD20. A) The weight of fetal rats on GD20, n = 12;B) Alcian blue and alizarin red staining of the fetal skeleton on GD20 (black arrow indicated the cartilage; red arrow indicated the ossification center); C) Representative Safranin O‐fast green staining of fetal cartilage (R, C, and H represent round, columnar and hypertrophic zones of cartilage); D) The quantification of MOD of Safranin O‐fast green staining, n = 5; E−G) The quantitative sizes of round, columnar and hypertrophic zones of cartilage, n = 5; H) RT‐qPCR was used to determine the mRNA expression of Col2a1, Acan, Mmp3, Mmp13 and Adamts5, n = 12; I−K) The protein expression and quantification (MOD) of Col2a1and Acan detected by immunohistochemical staining in fetal rat cartilage on GD20, n = 5. Values are expressed as the means ± S.E.M. ^*^ *P* <0.05, ^**^ *P* <0.01, ^***^ *P* <0.001 versus corresponding control. PPE, prenatal prednisone expression; GD, gestational day; CON, control; MOD, mean optical density; Col2a1, collagen type II alpha 1; Acan, aggrecan; Mmp3, matrix metallopeptidase 3; Mmp13, matrix metallopeptidase 13; Adamts5, ADAM metallopeptidase with thrombospondin type 1 motif 5; RT‐qPCR, real‐time quantitative polymerase chain reaction.

Previous studies have suggested that fetal‐derived chondrodysplasia is associated with low birth weight.^[^ ^15^ ^]^ Therefore, we first evaluated the effects of PPE on fetal body weight. The results showed that PPE decreased fetal rat body weight in a dose‐dependent manner (**Figure** **1**A). Alcian blue and alizarin red staining also indicated that a high dose (0.25 mg kg^−1^∙d) of PPE (PPE(H)) resulted in shortened body length, reduced limb cartilage, and delayed appearance of ossification centers in fetal rats (Figure 1B). Safranin O‐fast green staining to evaluate cartilage quality showed that, compared with the control group, both low doses (0.125 mg kg^−1^∙d) of PPE (PPE(L)) and PPE(H) caused uneven and shallower staining and decreased mean optical density (MOD) values. The areas of round (R) and columnar (C) chondrocyte layers in the proliferating zone of cartilage tissue were significantly reduced, whereas the hypertrophic (H) cell zone area was significantly increased in the PPE(L) and PPE(H) groups compared to the control. Furthermore, these changes were dose‐dependent (Figure 1C–G). Real‐time quantitative polymerase chain reaction (RT‐qPCR) results revealed that both PPE(L) and PPE(H) decreased the mRNA expression of the cartilage matrix synthesis‐related genes aggrecan (Acan) and collagen type II alpha 1 (Col2a1) as well as the degradation‐related genes matrix metallopeptidase (Mmp) 3, Mmp13, and ADAM metallopeptidase with thrombospondin type 1 motif 5 (Adamts5) (Figure 1H). Immunohistochemical staining also confirmed that PPE(H) decreased the protein expression of Acan and Col2a1 (Figure 1I–K). These results indicate that PPE can cause chondrodysplasia in fetal rats, which is associated with the inhibition of cartilage proliferation and extracellular matrix synthesis.

**Figure 2:**
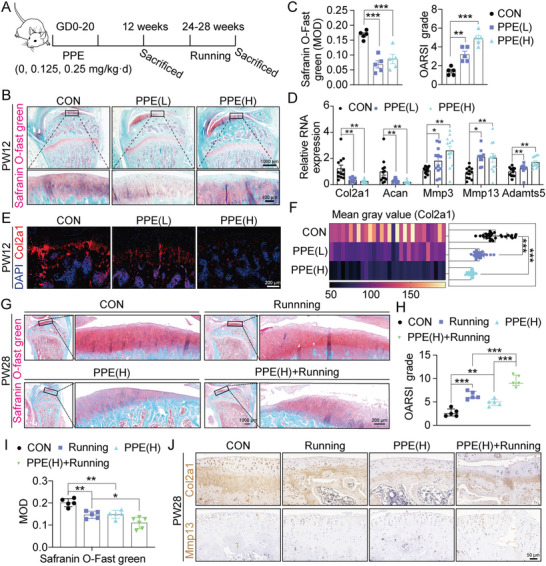
Effects of PPE on cartilage quality and OA susceptibility in adult offspring rats. A) Schemes of rats being treated; B) Safranin O‐fast green staining of rat cartilage at PW12, n = 5; C) The quantification (MOD) and OARSI score of Safranin O‐fast green staining at PW12, n = 5; D) The mRNA expression of Col2a1, Acan, Mmp3, Mmp13, and Adamts5 in cartilage at PW12 through RT‐qPCR, n = 12; E−F) The protein expression and quantification of Col2a1 detected by immunofluorescence, n = 5 × 6; G) Safranin O‐fast green staining of rat cartilage at PW28, n = 5; H) OARSI score of Safranin O‐fast green staining, n = 5; I) The quantification (MOD) of Safranin O‐fast green staining of rat cartilage at PW28, n = 5; J) The protein expression of Col2a1 and Mmp13 assayed by immunohistochemistry, n = 5. Values are expressed as the means ± S.E.M. ^*^ *P* <0.05, ^**^ *P* <0.01, ^***^ *P* <0.001 versus corresponding control. PPE, prenatal prednisone expression; OA, osteoarthritis; GD, gestational day; PW, postnatal week; CON, control; MOD, mean optical density; OARSI, Osteoarthritis Research Society International; Col2a1, collagen type II alpha 1; Acan, aggrecan; Mmp3, matrix metallopeptidase 3; Mmp13, matrix metallopeptidase 13; Adamts5, ADAM metallopeptidase with thrombospondin type 1 motif 5; RT‐qPCR, real‐time quantitative polymerase chain reaction.

To further investigate the effects of intrauterine chondrodysplasia caused by PPE on adult offspring, the progeny rats were raised after birth and subjected to long‐distance running during PW24–28 (**Figure** **2**A). At PW12, Safranin O‐fast green staining showed that PPE(L) and PPE(H) resulted in uneven and shallower staining, decreased MODs, thinner cartilage, structural disorder, tidemark interruption or even disappearance, and increased Osteoarthritis Research Society International (OARSI) scores compared to controls (Figure 2B,C). RT‐qPCR showed a dose‐dependent decrease in the mRNA expression of genes related to cartilage matrix synthesis (Acan and Col2a1) and a dose‐dependent increase in the mRNA expression of some genes related to cartilage matrix degradation (Mmp3 and Adamts5) with PPE treatment; although the dose‐dependency of the expression of another matrix degradation‐related gene Mmp13 was not significant, it was also significantly increased in the PPE(L) and PPE(H) groups compared to the control (Figure 2D). Immunofluorescence and immunohistochemical staining also confirmed a dose‐dependent decrease in the protein expression of Acan and Col2a1 induced by PPE (Figure 2E,F; Figure S1A,B, Supporting Information) and an increase in Mmp13 protein expression (Figure S1C,D, Supporting Information). At PW28, compared with the control, the PPE(H) and running‐only groups exhibited a shallower Safranin O‐fast green staining, decreased MODs, structural disorder, surface irregularities, increased OARSI scores (Figure 2G–I), decreased Acan and Col2a1 mRNA and protein expression (Figure 2J; Figure S1E–H, Supporting Information), and an increase in mRNA expression of Mmp3, Mmp13, and Adamts5 and protein expression of Mmp13 in different degrees (Figure 2J; Figure S1E–I, Supporting Information). Compared with the running‐only group, the PPE(H)+Running group showed more significant changes in the aforementioned indicators, and the cartilage surface exhibited typical OA‐like changes, including notable fibrosis and cracks (Figure 2G–J; Figure S1E–I, Supporting Information). Overall, PPE‐induced chondrodysplasia persisted after birth and increased susceptibility to OA in adult offspring.

### 2.2 circGtdc1 Participates in PPE‐Induced Chondrodysplasia in Offspring Rats

**Figure 3:**
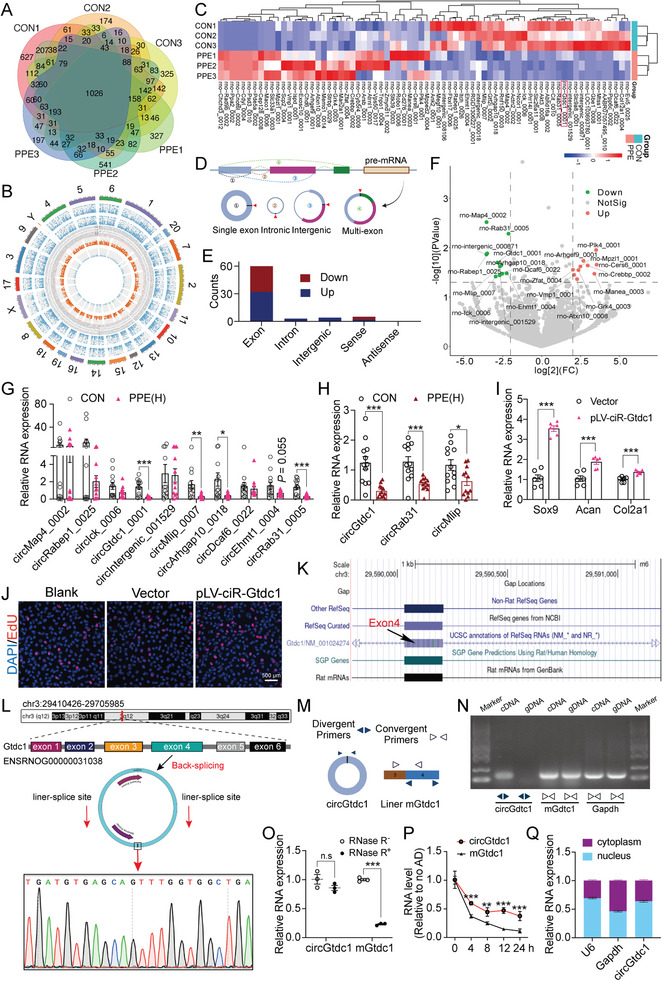
Effects of PPE on circRNomics in fetal cartilage on GD20. A) Venn diagram showing the expression of circRNA detected in each group; B) Circos plot indicating the differentially expressed circRNAs (the outermost circle shows the chromosomal distribution of the circRNAs; the second and third circle shows the expression levels of the indicated circRNAs; the fourth circle indicates the ID of the circRNAs; the fifth circle shows the lgFC of the indicated circRNAs between the two groups; the innermost circle shows the lg*P* values); C) Hotmap showing circRNAs differentially expressed between CON and PPE group; D) The classification of circRNA according to the origin; E) The counts of classification of circRNA differentially expressed between CON and PPE group; F) Volcano plot showing circRNAs differentially when |log_2_FC|>2 and *P* < 0.05; RT‐qPCR detected the circRNA expression in cartilage on GD20 G) and PW12 H) as indicated, n = 12; I) RT‐qPCR was used to assay the mRNA expression of Sox9, Acan, and Col2a1 in fetal chondrocytes treated with plasmid of circGtdc1 (pLV‐ciR‐circGtdc1), n = 3 × 2; J) EdU was used to assay the proliferation ability of fetal chondrocytes treated with pLV‐ciR‐Gtdc1, n = 3; K) circGtdc1 information from the UCSC Genome Browser; L) Schemes (upper) of formation and origin; Sanger sequencing (lower) confirmed the back‐splice junctions of circGtdc1; M,N) Divergent and convergent primers were used to verify the circGtdc1and mGtdc1 in gDNA and cDNA; O,P) circGtdc1 and mGtdc1 expression in chondrocytes with or without RNase R or actinomycin D (AD) treatment, n = 3; Q) circGtdc1, U6 and Gapdh expression in the nucleus and cytoplasm, n = 3. Values are expressed as the means ± S.E.M. ^*^ *P* <0.05, ^**^ *P* <0.01, ^***^ *P* <0.001 versus corresponding control. PPE, prenatal prednisone expression; GD, gestational day; CON, control; PW, postnatal week; circGtdc1, circular RNA Gtdc1; circRab31, circular RNA Rab31; circMlip, circular RNA Mlip; Sox9, SRY (sex‐determining region Y)‐box9; Col2a1, collagen type II alpha 1; Acan, aggrecan; EdU, 5‐ethynyl‐2′‐deoxyuridine; gDNA, genomic DNA; cDNA, complementary DNA; mGtdc1, mRNA of glycosyltransferase like domain containing 1; Gapdh, glyceraldehyde 3‐phosphate dehydrogenase; Gtdc1, glycosyltransferase like domain containing 1; AD, Actinomycin D; U6, U6 small nuclear RNA; FC, fold change; RT‐qPCR, real‐time quantitative polymerase chain reaction.

To investigate the underlying mechanisms of PPE‐induced chondrodysplasia and susceptibility to OA in offspring, circRNA sequencing was performed on cartilage samples collected on GD20. A total of 6199 circRNAs were detected in the cartilage, with 4914 and 4813 circRNAs in the control and PPE groups, respectively (**Figure** **3**A). These circRNAs, with lengths primarily ranging from 0 to 1000 nt, were distributed across all chromosomes (Figure 3B; Figure S2A, Supporting Information). Differential analysis further revealed 37 upregulated and 35 downregulated circRNAs in the PPE group compared with the control group (|log_2_FC|>1, *P* <0.05) (Figure 3B,C), most of which originated from gene exon sequences, with a small portion derived from introns and intergenic regions (Figure 3D,E; Figure S2B, Supporting Information). circRNAs with a |log_2_FC|>2 and *P*<0.05 were selected for further validation (Figure 3F). RT‐qPCR showed that circGtdc1_0001 (hereafter referred to as circGtdc1), circRab31_0005 (hereafter referred to as circRab31), and circMlip_0007 (hereafter referred to as circMlip) exhibited the most notable changes, which was consistent with the sequencing results, and showed the most significant statistical differences (Figure 3G; Figure S2C, Supporting Information).

Furthermore, the expression of these three circRNAs was examined in PPE‐treated rat cartilage at PW12 and PW28, revealing a significant decrease in PPE‐treated cartilage compared to controls (Figure 3H; Figure S2D, Supporting Information). Accordingly, we treated primary fetal rat chondrocytes with siRNAs targeting these circRNAs and observed a significant reduction in circGtdc1, circRab31, and circMlip expression after the respective specific siRNA treatment (Figure S2E–G, Supporting Information). Examination of gene expression related to cartilage matrix synthesis and degradation showed that circGtdc1 siRNA treatment significantly reduced SRY (sex‐determining region Y)‐box9 (Sox9), Acan, Col2a1, and Mmp3 mRNA expression without significantly impacting on Mmp13 and Adamts5 expression (Figure S2H, Supporting Information). circRab31 siRNA treatment decreased Sox9 and Acan expression and increased Mmp13 expression; however, it did not impact on Col2a1, Mmp3, and Adamts5 expression (Figure S2I, Supporting Information), while circMlip siRNA only reduced Mmp3 and Mmp13 mRNA expression (Figure S2J, Supporting Information). Hence, circGtdc1 knockdown had the most significant effect on the expression of genes related to chondrocyte matrix synthesis and degradation, which was consistent with the impact of PPE on cartilage tissue.

Subsequently, a plasmid overexpressing circGtdc1 (pLV‐ciR‐Gtdc1) was constructed (Figure S3A, Supporting Information) and its successful overexpression in primary fetal rat chondrocytes after transfection was confirmed using immunofluorescence (Figure S3B, Supporting Information). RT‐qPCR showed that pLV‐ciR‐Gtdc1 significantly increased circGtdc1 expression in chondrocytes, but had no significant effect on linear glycosyltransferase‐like domain containing 1 (Gtdc1) mRNA (mGtdc1) expression compared to controls (Figure S3C, Supporting Information). Meanwhile, RT‐qPCR and 5‐ethynyl‐2′‐deoxyuridine (EdU) assays showed that pLV‐ciR‐Gtdc1 significantly increased Sox9, Acan, and Col2a1 mRNA expression and promoted the proliferation of primary fetal rat chondrocytes compared to controls (Figure 3I,J; Figure S3D, Supporting Information). In summary, circGtdc1 was significantly downregulated in the offspring rat cartilage induced by PPE, and its knockdown or overexpression significantly impacted chondrocyte proliferation and matrix synthesis.

No previous studies have identified or characterized circGtdc1. Nevertheless, The CSC Genome Browser database showed that circGtdc1 is derived from exon 4 of Gtdc1 (Figure 3K). Hence, Sanger sequencing was used to validate the back‐splicing site (Figure 3L). To further verify the circGtdc1 structure, convergent and divergent primers (across the back‐splicing site) were designed to amplify circGtdc1 and linear mGtdc1, respectively (Figure 3M). Agarose gel electrophoresis showed that circGtdc1 was amplified only from cDNA, whereas mGtdc1 could be amplified from both cDNA and gDNA (Figure 3N), indicating that circGtdc1 is a product of back‐splicing of Gtdc1 pre‐mRNA. In addition, circGtdc1 was more resistant to RNase R (Figure 3O) and exhibited a longer half‐life than mGtdc1 (Figure 3P), as confirmed by an actinomycin D assay, indicating its higher stability. Furthermore, mGtdc1 could be amplified using Olig(dT) and random primers, whereas circGtdc1 failed to be reverse‐transcribed using Olig(dT), further confirming the circular structure of circGtdc1 without a poly‐A tail (Figure S3E, Supporting Information). Finally, nucleocytoplasmic fractionation experiments were performed, as the subcellular localization of circRNA is often associated with its function.^[^ ^25^ ^]^ The results showed the presence of circGtdc1 in both the nucleus and cytoplasm, with primary nuclear localization (Figure 3Q). Additionally, due to the non‐compliance of the circGtdc1 back‐splicing site with the principles of the fluorescence in situ hybridization (FISH) probe design, its subcellular localization could not be further verified using FISH. Collectively, these results suggest that circGtdc1 is a newly identified circRNA that may be involved in PPE‐induced chondrodysplasia.

### 2.3 Prednisolone Induces Changes in Fetal Chondrocyte Proliferation and Matrix Synthesis via circGtdc1

**Figure 4:**
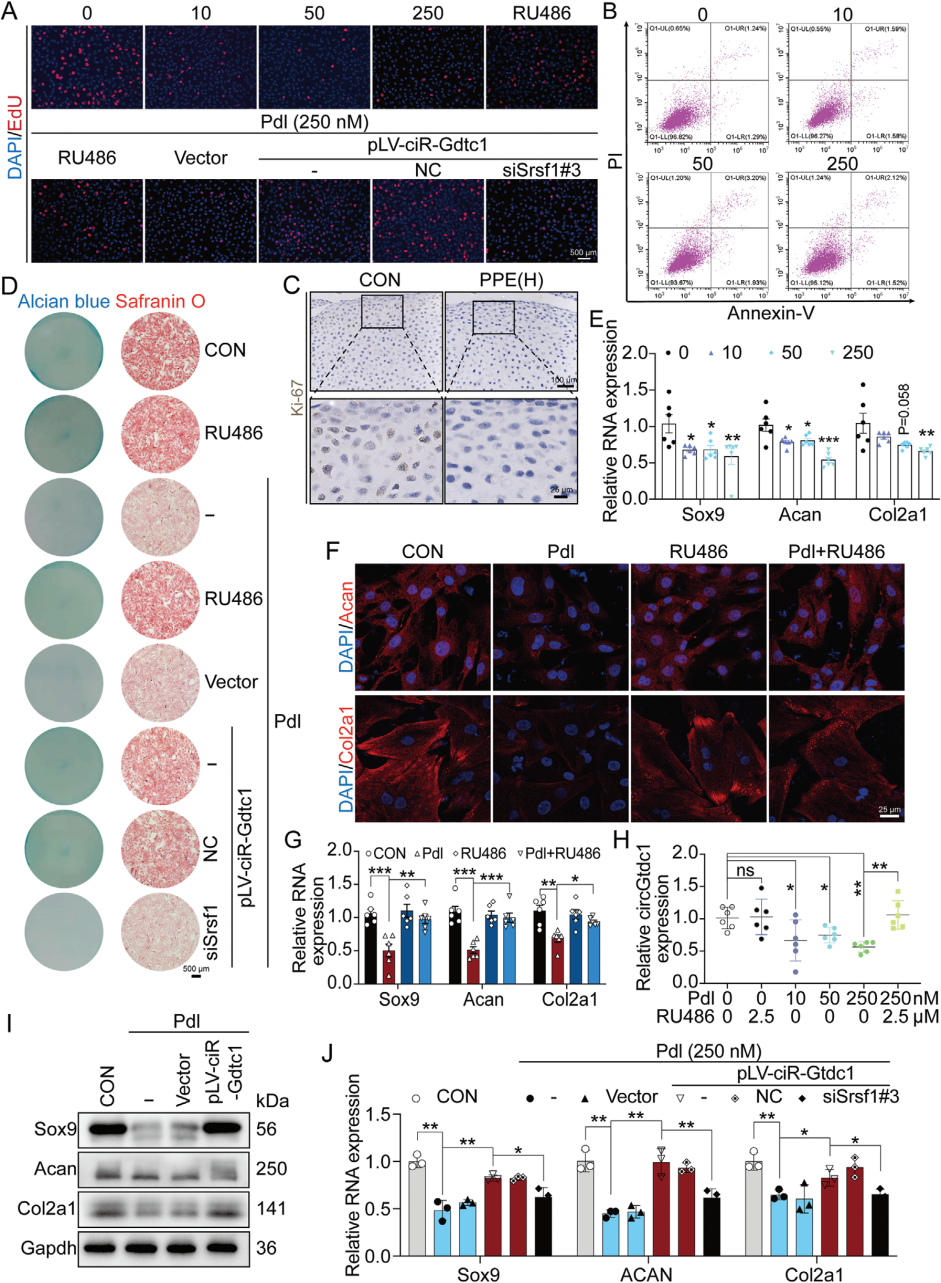
Effects of circGtdc1 on chondrocyte proliferation and extracellular matrix synthesis inhibited by prednisolone. A) EdU was used to assay the proliferation ability of fetal rat primary chondrocytes treated as indicated, n = 3; B) Flow cytometry was used to assay apoptosis of chondrocytes, n = 3; C) Immunohistochemistry was applied to assay the protein expression of Ki‐67 in cartilage on GD20, n = 5; D) Safranin O (n = 6) and Alcian blue (n = 5) staining were used to assay matrix contents of chondrocytes treated as indicated; E) RT‐qPCR was applied to confirm the mRNA expression of Sox9, Acan and Col2a1, n = 6; F) Immunofluorescence was used to confirm the protein expression of Acan and Col2a1, n = 6; G) RT‐qPCR was applied to detect the mRNA expression of Sox9, Acan, and Col2a1, n = 6; H) RT‐qPCR was used to detect the circGtdc1 expression in chondrocytes treated by prednisolone combined with or without RU486, n = 6; I) Western blotting was used to assay the protein expression of Sox9, Acan and Col2a1 in fetal rat chondrocytes induced by prednisolone (250 nm) combined with or without pLV‐ciR‐Gtdc1 plasmid, n = 3; J) RT‐qPCR was used to detect the mRNA expression of Sox9, Acan and Col2a1 in prednisolone‐induced chondrocytes treated by pLV‐ciR‐Gtdc1 combined with or without siRNA of Srsf1, n = 3. Values are expressed as the means ± S.E.M. ^*^ *P* <0.05, ^**^ *P* <0.01, ^***^ *P* < 0.001 versus corresponding control. circGtdc1, circular RNA Gtdc1; EdU, 5‐ethynyl‐2′‐deoxyuridine; Pdl, prednisolone; NC, negative control; Gtdc1, glycosyltransferase like domain containing 1; Srsf1, serine and arginine rich splicing factor 1; IHC, immunohistochemistry; Ki‐67, marker of proliferation Ki‐67; CON, control; PPE, prenatal prednisone expression; Sox9, SRY (sex‐determining region Y)‐box9; Col2a1, collagen type II alpha 1; Acan, aggrecan; Gapdh, glyceraldehyde 3‐phosphate dehydrogenase; RT‐qPCR, real‐time quantitative polymerase chain reaction.

To investigate the mechanisms by which PPE caused chondrodysplasia, the serum levels of prednisone and its active metabolite, prednisolone, were measured, which ranged from 2.1 to 50.8 and 4.1 to 57.2 nm, respectively (Table S1, Supporting Information). After treating fetal rat primary chondrocytes with different concentrations of prednisone, no significant effect was observed on proliferation, apoptosis, or matrix synthesis (Figure S4A–F, Supporting Information). After treatment with various concentrations of prednisolone (10, 50, and 250 nm), EdU staining indicated reduced fetal rat chondrocyte proliferation in a concentration‐dependent manner (**Figure** **4**A; Figure S5A, Supporting Information), but no significant effect on chondrocyte apoptosis was observed using flow cytometry (Figure 4B; Figure S5B, Supporting Information). Immunohistochemical staining confirmed that PPE reduced the protein expression of the proliferation‐associated protein Ki‐67 in fetal rat cartilage tissue (Figure 4C; Figure S5C, Supporting Information). Safranin O and Alcian blue staining revealed that prednisolone significantly reduced the matrix content of chondrocytes (Figure 4D; Figure S5D, Supporting Information). RT‐qPCR also showed that prednisolone concentration‐dependently reduced the expression of genes related to chondrocyte matrix synthesis (Sox9, Acan, and Col2a1) (Figure 4E), and immunofluorescence further confirmed that prednisolone decreased the protein expression of Acan and Col2a1 (Figure 4F; Figure S5E, Supporting Information). Considering that prednisolone is a synthetic glucocorticoid that exerts biological effects through the glucocorticoid receptor (GR),^[^ ^26^ ^]^ we treated fetal chondrocytes with prednisolone combined with the GR inhibitor RU486. RU486 treatment reversed all of the aforementioned changes caused by prednisolone (Figure 4A–G; Figure S5A–E, Supporting Information). Therefore, prednisolone (rather than prednisone) reduced the proliferation and matrix synthesis of chondrocytes in a concentration‐dependent manner.

Next, we investigated the effects of prednisolone on circGtdc1 expression. RT‐qPCR showed that prednisolone reduced the expression of circGtdc1 in a concentration‐dependent manner without significantly impacting on mGtdc1 expression, which was reversed by RU486 treatment (Figure 4H; Figure S5F, Supporting Information). circRNA biogenesis depends on back‐splicing, and eukaryotic translation initiation factor 4A3 (eIF4A3) is a core component of the exon junction complex and plays an essential role in pre‐mRNA splicing.^[^ ^27^ ^]^ catRAPID and RPISeq show that eIF4A3 can bind to the flanking regions of circGtdc1, suggesting that it can regulate circGtdc1 expression.^[^ ^27^ ^]^ JASPAR also suggests that eIF4A3 promoter contains glucocorticoid response elements. Therefore, eIF4A3 level was assayed and the results showed that PPE and prednisolone decreased the mRNA and protein expression of eIF4A3 in cartilage tissue or chondrocytes, respectively, while RU486 reversed the effects of prednisolone (Figure S5G–J, Supporting Information). Moreover, eIF4A3 knockdown via siRNAs also decreased circGtdc1 expression, whereas eIF4A3 overexpression reversed the decreased circGtdc1 induced by prednisolone (Figure S5K,L, Supporting Information), suggesting that eIF4A3 mediates the prednisolone‐induced decrease in circGtdc1. Furthermore, we treated prednisolone‐induced primary chondrocytes with the circGtdc1 overexpression plasmid pLV‐ciR‐Gtdc1. EdU indicated that pLV‐ciR‐Gtdc1 significantly reversed the inhibitory effects of prednisolone on chondrocyte proliferation (Figure 4A; Figure S5A, Supporting Information). Safranin O staining, Alcian blue staining, RT‐qPCR, and western blotting demonstrated that pLV‐ciR‐Gtdc1 reversed the decrease in mRNA and protein expression of Sox9, Acan, and Col2a1, as well as the matrix content induced by prednisolone in primary fetal rat chondrocytes (Figure 4D–J; Figure S5D–M). Collectively, these results suggest that the prednisolone‐induced decrease in circGtdc1 via eIF4A3 inhibits the proliferation and matrix synthesis of fetal rat chondrocytes.

### 2.4 circGtdc1 Interacts with Srsf1 Protein and Inhibits its Degradation via K48‐Linked Polyubiquitination

**Figure 5:**
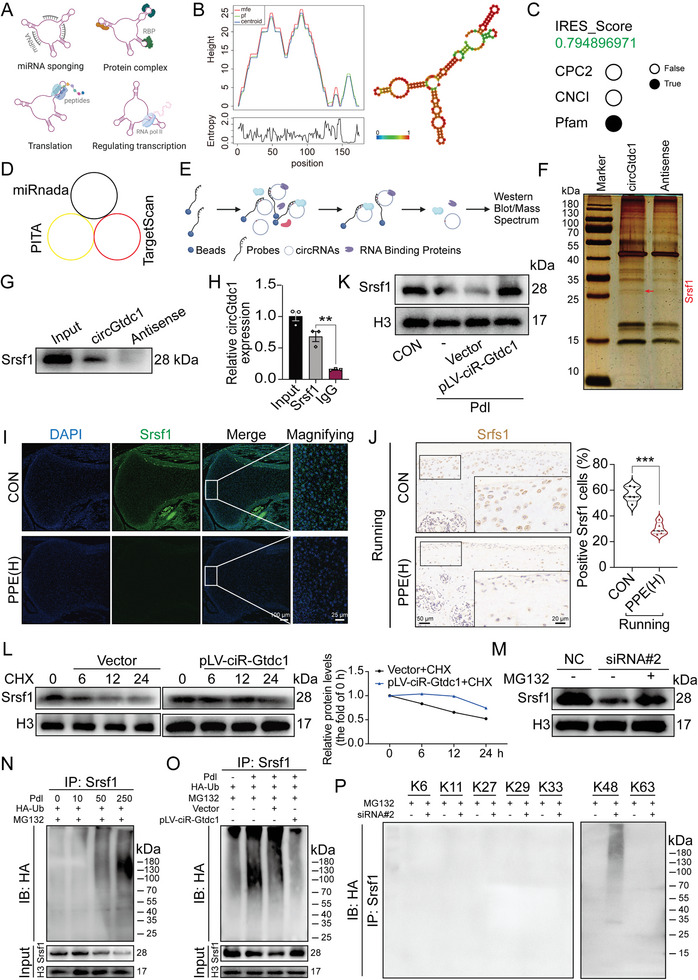
Influences of circGtdc1 on Srsf1 protein stability. A) circRNA functions; B) The predicted mountain plot representation of the thermodynamic ensemble (left) and secondary structure (right) of circGtdc1 based on minimum free energy by *RNAfold* WebServer; C) The IRES score and coding potential predicted by Coding Potential Calculator 2 (CPC2), Coding‐Non‐Coding Index (CNCI), and Pfam software; D) The binding potential of circGtdc1 to miRNAs was predicted by miRnada, PITA and Targetscan software from circAtlas 2.0 database; E) Schemes of RNA pulldown assay; F) Silver staining of proteins from RNA pulldown assay by the specific biotin‐labeled circGtdc1 probe in rat primary chondrocytes; G) Western blotting was performed to detect Srsf1 protein in samples from RNA pulldown assay; H) RIP assay was used to detect the binding between circGtdc1 and Srsf1, n = 3; I) Immunofluorescence was used to confirm the protein level of Srsf1 in fetal rat cartilage on GD20, n = 5; J) Immunohistochemistry was applied to assay the protein level of Srsf1 in cartilage at PW28, n = 5; K) Western blotting was performed to detect Srsf1 protein levels in chondrocytes treated by prednisolone (250 nm) combined with or without pLV‐ciR‐Gtdc1, n = 3; L) Srsf1 protein level in fetal primary chondrocytes with circGtdc1 overexpression treated with CHX (50 µg mL^−1^) for 0, 6, 12, or 24 h; M) The Srsf1 protein level in fetal primary chondrocytes with circGtdc1 knockdown treated with MG132 (20 µM) for 6 h; N) The ubiquitination of Srsf1 proteins in fetal primary chondrocytes treated with prednisolone (0, 10, 50, and 250 µm); the proteins precipitated in the IP assay were analyzed by Western blotting; O) Western blotting and IP showing the effects of circGtdc1 overexpression on the ubiquitination of Srsf1 protein in fetal rat primary chondrocytes induced by prednisolone (250 µm); P) Western blotting and IP assay showing the upregulation of K48‐linked ubiquitination in circGtdc1‐knochdown chondrocytes (K6, K11, K27, K29, K33, K48, and K63 represent the plasmids expressing HA‐tagged ubiquitin with all lysine mutated except themselves). Values are expressed as the means ± S.E.M. ^*^ *P* <0.05, ^**^ *P* <0.01, ^***^ *P* <0.001 versus corresponding control. circGtdc1, circular RNA Gtdc1; Srsf1, serine and arginine rich splicing factor 1; miRNA, microRNA; IRES, internal ribosome entry site; IgG, immunoglobulin G; CON, control; PPE, prenatal prednisone expression; IHC, immunohistochemistry; Pdl, prednisolone; CHX, cycloheximide; NC, negative control; Ub, ubiquitin; IB: immunoblot; HA, hyaluronic acid; IP: immunoprecipitation; RIP, RNA immunoprecipitation.

circRNAs often exert their biological functions through various mechanisms,^[^ ^28^ ^]^ including microRNA (miRNA) sponges, translating peptides, binding to proteins, or regulating parental gene transcription (**Figure** **5**A). To investigate the molecular mechanisms of circGtdc1, we first predicted its secondary structure using RNAfold, revealing three stem‐loop structures in circGtdc1 with minimum free energy (Figure 5B). Meanwhile, although the circAtlas 2.0 database showed an internal ribosome entry site (IRES) score of >0.5 for circGtdc1 (0.79), it did not possess an open reading frame. Moreover, both CPC2 and CNCI software predicted that circGtdc1 lacked the potential to encode peptides, excluding Pfam (Figure 5C). Additionally, although miRnada, PITA, and TargetScan predicted that circGtdc1 could bind to miRNAs, there was no overlap in the predictions from the three software (Figure 5D). RNA immunoprecipitation (RIP) assay also indicated no interaction between circGtdc1 and argonaute 2 (AGO2) protein (Figure S6A,B, Supporting Information), which is necessary for miRNA sponging.^[^ ^29^ ^]^ Furthermore, PPE, prednisolone, and the circGtdc1 knockdown or overexpression did not alter the expression of its parental gene, mGtdc1, in cartilage tissue or chondrocytes (Figures S3C, S5F, and S6C,D, Supporting Information). Therefore, circGtdc1 likely does not exert its biological functions through miRNA sponges, translating peptides, or regulating parental gene transcription.

Subsequently, we investigated whether circGtdc1 binds to RNA‐binding proteins by RNA pulldown combined with mass spectrometry (Figure 5E). Silver staining and mass spectrometry suggested that multiple proteins bound to circGtdc1 (Figure 5F; Figure S6E, Supporting Information). Kyoto Encyclopedia of Genes and Genomes (KEGG) and STRING analyses further revealed that these proteins mainly regulated RNA splicing, with serine‐ and arginine‐rich splicing factor 1 (Srsf1) located at the center of these interactions (Figure S6E,F, Supporting Information). Moreover, RPISeq showed that circGtdc1 physiologically interacts with Srsf1. RIP and RNA pulldown confirmed the interaction between circGtdc1 and Srsf1 (Figure 5G,H). Therefore, we examined the effects of PPE and prednisolone on Srsf1 expression. PPE did not alter Srsf1 mRNA expression in cartilage tissues (Figure S6G, Supporting Information); however, it significantly decreased Srsf1 protein levels in the cartilage at GD20, PW12, and PW28 after running (Figure 5I,J; Figure S6H,I, Supporting Information). In vitro, prednisolone also did not alter Srsf1 mRNA expression (Figure S6J, Supporting Information) but decreased Srsf1 protein levels in a concentration‐dependent manner (Figure S6K,L, Supporting Information). Furthermore, circGtdc1 siRNA decreased Srsf1 protein expression in chondrocytes, whereas pLV‐ciR‐Gtdc1 reversed the decrease in Srsf1 protein levels in chondrocytes induced by prednisolone (Figure 5K; Figure S6M,N, Supporting Information). Moreover, the cycloheximide (CHX) assay showed that pLV‐ciR‐Gtdc1 increased the half‐life of Srsf1 (Figure 5L). Taken together, these results demonstrate that circGtdc1 binds to Srsf1 and mediates its degradation induced by prednisolone.

Protein degradation is primarily regulated by the autophagy‐lysosome pathway (ALP) and ubiquitin‐proteasome system (UPS).^[^ ^30^ ^]^ Therefore, we treated fetal chondrocytes with the ALP inhibitor bafilomycin A1 (BafA1)^[^ ^31^ ^]^ in combination with circGtdc1 siRNA. Western blotting results showed that BafA1 failed to reverse circGtdc1 siRNA‐induced degradation of the Srsf1 protein (Figure S6O, Supporting Information), indicating that circGtdc1 does not regulate Srsf1 degradation via autophagy. Next, we treated chondrocytes with the proteasome inhibitor MG132^[^ ^32^ ^]^ to determine whether circGtdc1 regulates Srsf1 degradation via the UPS. The results showed that MG132 significantly inhibited the degradation of the Srsf1 protein induced by circGtdc1 siRNA (Figure 5M). Subsequently, the ubiquitination level of Srsf1 was assayed using immunoprecipitation (IP). The results showed that prednisolone increased the ubiquitination level of Srsf1 in a concentration‐dependent manner, whereas circGtdc1 overexpression reversed this effect (Figure 5N,O). Protein ubiquitination chains primarily include K6, K11, K27, K29, K33, K48, and K63.^[^ ^30^ ^]^ Therefore, chondrocytes were treated with corresponding plasmids in combination with circGtdc1 siRNA. The results showed that circGtdc1 downregulation primarily regulated the K48‐linked polyubiquitination of Srsf1 (Figure 5P). Together, these results indicate that prednisolone primarily promotes the K48‐linked polyubiquitin of the Srsf1 protein through circGtdc1, thus inducing its degradation.

Ubiquitylation begins when ubiquitin is activated by ubiquitin‐activating enzymes (E1). The activated ubiquitin is then transferred to ubiquitin‐conjugating enzymes (E2). Finally, E3 ligases tag the activated ubiquitin from E2‐ ubiquitin complex to the substrates.^[^ ^33^ ^]^ Ubiquitin ligases (E3) convey ubiquitin substrate specificity.^[^ ^33^ ^]^ Therefore, we further investigated the E3 ligases. Previous studies showed that parkin RBR E3 ubiquitin‐protein ligase (PARK2)^[^ ^34^ ^]^ and ring finger protein 125 (RNF125)^[^ ^35^ ^]^ can regulate Srsf1 ubiquitination in human lung cancer and liver cancer cells. UbiBrowser also predicted that beta‐transducin repeat containing E3 ubiquitin protein (BTRC) is the Srsf1 specific E3 ligase (Figure S7A, Supporting Information). Therefore, the binding of PARK2, RNF125, and BTRC to Srsf1 protein was detected by Co‐IP and the results showed that only BTRC could bind Srsf1 protein in rat primary chondrocytes (Figure S7B, Supporting Information). RT‐qPCR, western blotting, and immunofluorescence suggested that prednisolone did not alter the mRNA and protein expression of BTRC, but significantly promoted its binding to Srsf1 (Figure S7C–E, Supporting Information). Knocking down BTRC inhibited the increased ubiquitination of Srsf1 after circGtdc1 siRNA treatment, while BTRC overexpression significantly reversed the reduction of Srsf1 ubiquitination caused by pLV‐ciR‐Gtdc1 in prednisolone‐induced chondrocytes (Figure S7F–H, Supporting Information). These results indicate that the reduction of circGtdc1 caused by prednisolone promotes the binding of BTRC to Srsf1 protein, thereby inducing Srsf1 ubiquitination.

### 2.5 Low Levels of Srsf1 Mediated Chondrocyte Proliferation and Extracellular Matrix Synthesis Inhibition Caused by Prednisolone by Inhibiting EDA/B+Fn1 Formation

**Figure 6:**
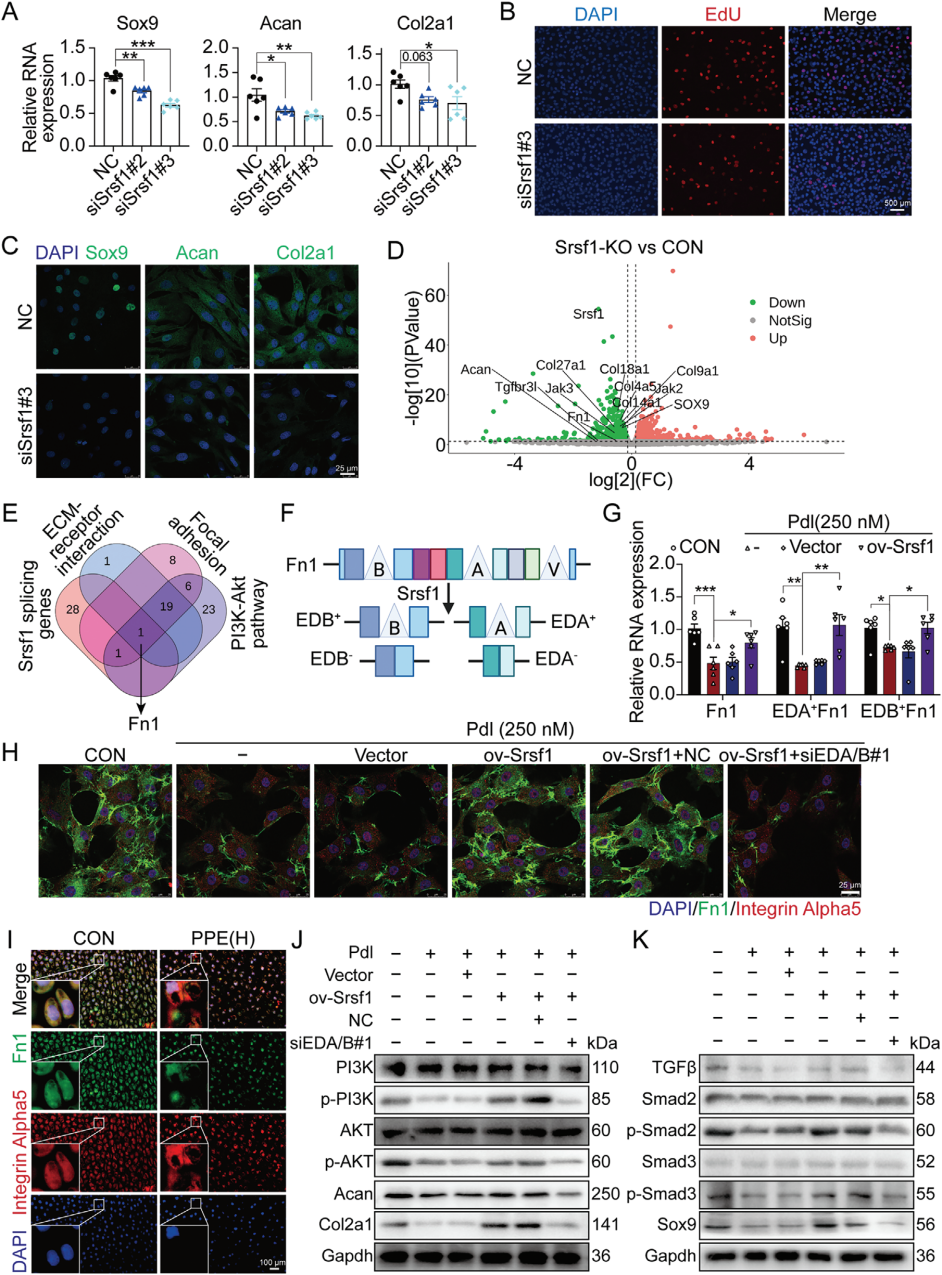
Influences and mechanism of Srsf1 in chondrocyte proliferation and extracellular matrix synthesis. A) RT‐qPCR was used to detect the mRNA expression of Sox9, Col2a1 and Acan in Srsf1‐knockdown chondrocytes, n = 6; B) EdU was used to assay the proliferation ability of fetal rat chondrocytes treated by Srsf1 siRNA, n = 6; C) The protein expression of Sox9, Col2a1 and Acan assayed by immunofluorescence in primary fetal rat chondrocytes treated by Srsf1 siRNA, n = 6; D) Volcano plot showing changes in mRNA expression by RNA sequencing; E) Venn diagram showing the collective gene in the top three KEGG pathway by RNA sequencing and Srsf1 splicing genes predicted by miasDB database; F) EDA^+^ and EDB^+^ Fn1 formation; G) RT‐qPCR detected the RNA expression of Fn1, EDA^+^ and EDB^+^ Fn1 in fetal primary chondrocytes with Srsf1 overexpression induced by prednisolone (250 nM), n = 6; H) Immunofluorescence was performed to assay the colocalization between Fn1 and integrin alpha 5 in fetal primary chondrocytes induced by prednisolone (250 nm) combined with Srsf1 overexpression and DA/B^+^Fn1 deletion, n = 5; I) Immunofluorescence was performed to assay the colocalization between Fn1 and integrin alpha 5 in rat cartilage tissue on GD20, n = 5; J,K) Western blotting was used to detect the protein expression of PI3K, p‐PI3K, AKT, p‐AKT, Acan, Col2a1, TGFβ, Smad2, p‐Smad2, Smad3, p‐Smad3, and Sox9 in prednisolone (250 nm)‐induced fetal primary chondrocytes treated with Srsf1 overexpression plasmid combining with or without EDA/B^+^Fn1 siRNA, n = 3. Values are expressed as the means ± S.E.M. ^*^ *P* <0.05, ^**^ *P* <0.01, ^***^ *P* <0.001 versus corresponding control. Srsf1, serine and arginine rich splicing factor 1; NC, negative control; Sox9, SRY (sex‐determining region Y)‐box9; Col2a1, collagen type II alpha 1; Acan, aggrecan; EdU, 5‐ethynyl‐2′‐deoxyuridine; CON, control; PI3K, phosphoinositide 3‐kinase; AKT, AKT serine/threonine kinase; Fn1, fibronectin 1; Pdl, prednisolone; PPE, prenatal prednisone expression; p‐PI3K, phospho‐phosphoinositide 3‐kinase; p‐AKT, phospho‐AKT; TGFβ, transforming growth factor beta; Smad2, SMAD family member 2; p‐Smad2, phospho‐SMAD family member 2; Smad3, SMAD family member 3; p‐Smad3, phospho‐SMAD family member 2; Gapdh, glyceraldehyde‐3‐phosphate dehydrogenase; RT‐qPCR, real‐time quantitative polymerase chain reaction.

To investigate the role of Srsf1 in chondrocytes, three Srsf1 siRNAs were used to treat primary fetal rat chondrocytes. The results showed that siSrsf1#2 and siSrsf1#3 significantly reduced Srsf1 mRNA and protein expression (Figure S8A, Supporting Information). RT‐qPCR, EdU, and immunofluorescence indicated that Srsf1 knockdown significantly decreased chondrocyte proliferation and the mRNA and protein expression of genes related to cartilage matrix synthesis (Sox9, Acan, and Col2a1) (**Figure** **6**A–C; Figure S8B,C, Supporting Information). Next, we investigated whether Srsf1 mediates the changes in chondrocyte proliferation and matrix synthesis induced by prednisolone via circGtdc1 in fetal chondrocytes. Prednisolone‐treated chondrocytes were treated with pLV‐ciR‐Gtdc1 combined with Srsf1 siRNA. EdU showed that Srsf1 knockdown inhibited the proliferation increased by circGtdc1 overexpression in prednisolone‐treated chondrocytes (Figure 4A; Figure S5A, Supporting Information). RT‐qPCR, Safranin O, and Alcian blue staining also showed that Srsf1 knockdown inhibited the circGtdc1‐caused increase in the mRNA expression of genes related to matrix synthesis (Sox1, Acan, and Col2a1) and extracellular matrix content in prednisolone‐treated chondrocytes (Figure 4D–J; Figure S5D, Supporting Information). These results suggest that circGtdc1‐Srsf1 signaling mediates the prednisolone‐induced decrease in chondrocyte proliferation and extracellular matrix synthesis.

KEGG analysis of proteins identified via mass spectrometry following RNA pulldown indicated that circGtdc1 binding proteins primarily regulate gene splicing (Figure S6F, Supporting Information) and that Srsf1 is crucial for alternative RNA splicing.^[^ ^36^ ^]^ MiasDB was then used to predict its downstream targets, revealing that Srsf1 regulates the alternative splicing of multiple genes (Figure S8D, Supporting Information); however, we detected no changes in the expression of common genes related to the regulation of cartilage proliferation and matrix synthesis, namely, calcium/calmodulin‐dependent protein kinase (CaM Kinase) II delta (CaMK2δ), CAMP responsive element binding protein (Creb), and Rac family small GTPase 1 (Rac1), in prednisone‐induced chondrocytes or following circGtdc1 overexpression or knockout (Figure S8E, Supporting Information). Therefore, we knocked out Srsf1 in the chondrocytes and performed RNA sequencing. The volcano plots showed that the expression of Srsf1 and genes related to matrix synthesis (Sox9, Acan, fibronectin 1 (Fn1), collagen type IV alpha 5 chain (Col4a5), Col9a1, Col14a1, and Col27a1) was significantly reduced, whereas CaMK2δ, Creb, and Rac1 expression did not markedly change in Srsf1‐knockout chondrocytes (Figure 6D), which further verified the reliability of the sequencing and above results. KEGG analysis revealed that Srsf1 primarily regulated the extracellular matrix‐receptor interaction, focal adhesion, and phosphoinositide 3‐kinase (PI3K)/AKT serine/threonine kinase (AKT) signaling pathways (Figure S8F, Supporting Information). Combining these pathways with the MiasDB prediction, we identified a single common gene, Fn1 (Figure 6E). The prediction results also indicated that Srsf1 primarily regulated the alternatively spliced forms of EDA/B^+^Fn1 (Figure 6F). Therefore, the expression of total Fn1 and EDA/B^+^Fn1 subtypes was detected. The results showed that the expression of total Fn1 mRNA and protein, as well as EDA/B^+^Fn1 subtypes, was significantly reduced in the cartilage tissue of the PPE(H) group at GD20 and PW28, as well as in chondrocytes treated with Srsf1 siRNA or prednisolone, while the in vitro overexpression of Srsf1 reversed the effects of prednisolone (Figure 6G–I; Figures S8G–I and S9A–D, Supporting Information). This suggests that Srsf1 mediates changes in Fn1 expression and alternative splicing induced by prednisolone.

Fn1 primarily binds to integrin receptors through extracellular matrix‐receptor interaction in cartilage, thereby regulating the PI3K/AKT and TGFβ pathways associated with chondrocyte proliferation and matrix synthesis.^[^ ^37^ ^]^ RNA sequencing following Srsf1 knockdown also revealed changes in the PI3K/AKT pathway (Figure S8F, Supporting Information). Therefore, we further examined the interaction between Fn1 and integrin receptors and changes in the PI3K/AKT and TGFβ pathways. Immunofluorescence and western blotting showed that prednisolone significantly reduced the colocalization of Fn1 with integrin receptor α5 (ITGα5) and the protein expression of phospho‐PI3K (p‐PI3K), phospho‐AKT (p‐AKT), TGFβ, phospho‐SMAD family member (p‐Smad) 2, p‐Smad3, and Sox9 in fetal chondrocytes, but did not affect the expression of PI3K, AKT, Smad2, and Smad3 (Figure 6H–K; Figure S9D–F, Supporting Information). Immunohistochemistry and fluorescence also showed a significant decrease in the colocalization of Fn1 with ITGα5 and the protein expression of p‐PI3K, p‐AKT, p‐Smad2, and Sox9 in the cartilage of the PPE(H) group at GD20 and PW28 (Figure 6I; Figures S9A–G and S10A, Supporting Information). To further verify whether the Srsf1‐EDA/B^+^Fn1 axis mediated the effects of prednisolone, fetal chondrocytes treated with prednisolone were induced to overexpress Srsf1 in combination with EDA/B^+^Fn1 siRNA (Figure S10B,C, Supporting Information). The results showed that Srsf1 overexpression reversed the decrease in the expression of EDA/B^+^Fn1 subtypes, Fn1 protein, and the colocalization of Fn1 with ITGα5, as well as p‐PI3K, p‐AKT, TGFβ, p‐Smad2, p‐Smad3, and Sox9 proteins, induced by prednisolone, while EDA/B^+^Fn1 siRNA effectively canceled these effects (Figure 6G–K; Figure S9D–F, Supporting Information). EdU, RT‐qPCR, and western blotting further confirmed the effects on chondrocyte proliferation and matrix synthesis, showing that Srsf1 overexpression partially reversed the inhibition of chondrocyte proliferation and the decrease in the expression of Sox9, Acan, and Col2a1 mRNA and proteins induced by prednisolone; EDA/B^+^Fn1 knockout also canceled these effects (Figure 6J–K; Figures S9E,F and S10D–F, Supporting Information). In summary, the decrease in Srsf1 caused a reduction in EDA/B^+^Fn1, which mediated the inhibition of chondrocyte proliferation and extracellular matrix synthesis induced by prednisolone.

### 2.6 circGtdc1 Reverses Chondrodysplasia Induced by PPE by Regulating Srsf1‐Fn1 Signaling In Vivo

**Figure 7:**
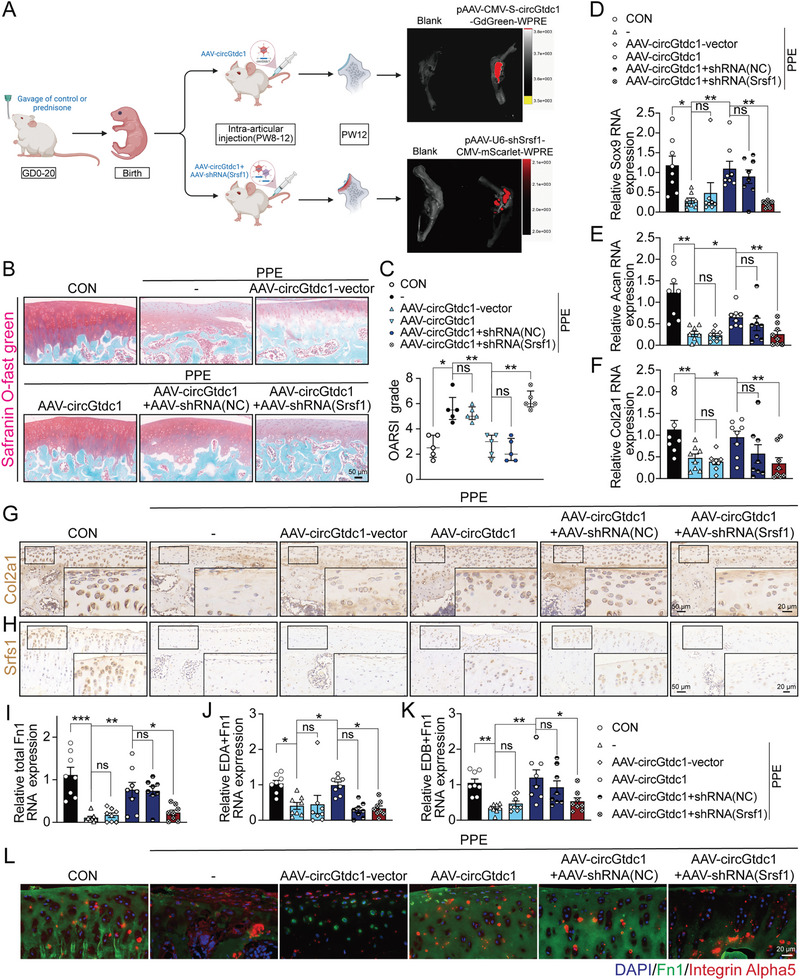
Influences of circGtdc1 overexpression combined with or without Srsf1 knockdown in rat cartilage induced by PPE. A) Schematic diagram and in vivo bioluminescence imaging of intraarticular injection of adeno‐associated virus (AAV) encapsulated with circGtdc1 plasmid (AAV‐circGtdc1) and/or Srsf1 shRNA (AAV‐shRNA(Srsf1)) in rats induced by PPE; B) Safranin O‐fast green staining of cartilage in PPE‐induced rats treated with AAV‐circGtdc1 and/or AAV‐shRNA(Srsf1) during PW8‐12, n = 5; C) OARSI score of Safranin O‐fast green staining, n = 5; D–F) RT‐qPCR was used to detect the mRNA expression of Sox9, Col2a1, and Acan, n = 8; G,H) Immunohistochemistry was applied to assay the protein level of Col2a1 and Srsf1 in cartilage treated as indicated, n = 5; I–K) RT‐qPCR was used to detect the mRNA expression of Fn1, EDA^+^Fn1, and EDB^+^Fn1, n = 8; L) Immunofluorescence was performed to assay the colocalization between Fn1 and integrin alpha 5 in cartilage tissue of rats treated as indicated, n = 5. Values are expressed as the means ± S.E.M. ^*^ *P* <0.05, ^**^ *P* <0.01, ^***^ *P* <0.001 versus corresponding control. circGtdc1, circular RNA Gtdc1; Srsf1, serine and arginine rich splicing factor 1; PPE, prenatal prednisone expression; GD, gestational day; PW, postnatal week; CON, control; AAV, adeno‐associated virus; NC, negative control; OARSI, Osteoarthritis Research Society International; Sox9, SRY (sex‐determining region Y)‐box9; Acan, aggrecan; Col2a1, collagen type II alpha 1; Fn1, fibronectin 1; RT‐qPCR, real‐time quantitative polymerase chain reaction.

Finally, we administered an adeno‐associated virus carrying the circGtdc1 plasmid (AAV‐circGtdc1) and Srsf1 shRNA (AAV‐shRNA(Srsf1)) via intra‐articular injection to PPE‐induced offspring rats during PW8–12, aiming to further verify the mechanisms of PPE‐induced poor cartilage quality and evaluate the therapeutic effect of circGtdc1 intervention (**Figure** **7**A). In vivo, bioluminescence imaging confirmed the successful expression of AAV‐circGtdc1 and AAV‐shRNA(Srsf1) in the rat joints (Figure 7A). Safranin O‐fast green staining showed that compared to the PPE group, intra‐articular injection of AAV‐circGtdc1 resulted in increased cartilage thickness, intensified staining, a smoother cartilage surface, and reduced OARSI scores; however, co‐administration of AAV‐shRNA(Srsf1) with AAV‐circGtdc1 nullified these effects (Figure 7B,C; Figure S11A, Supporting Information). Furthermore, RT‐qPCR and immunohistochemistry demonstrated that AAV‐circGtdc1 upregulated the mRNA expression of Sox9, Acan, and Col2a1, as well as the protein expression of Acan and Col2a1; similarly, AAV‐shRNA(Srsf1) effectively reversed these actions of AAV‐circGtdc1 (Figure 7D–G; Figure S11B–D, Supporting Information). Subsequently, we investigated the effects of AAV‐circGtdc1 on the Srsf1‐Fn1 axis. Immunohistochemistry and RT‐qPCR revealed that AAV‐circGtdc1 reversed the PPE‐induced reduction in Srsf1 protein levels, total Fn1 mRNA, and EDA/B^+^Fn1 expression in the cartilage tissue (Figure 7H–K; Figure S11E, Supporting Information); immunofluorescence analysis further substantiated that AAV‐circGtdc1 augmented Fn1 protein expression and its colocalization with ITGα5, whereas the combined administration of AAV‐shRNA(Srsf1) and AAV‐circGtdc1 also abrogated above these effects (Figure 7H–L; Figure S11E–G, Supporting Information). Therefore, it is suggested that the in vivo overexpression of circGtdc1 could ameliorate PPE‐induced chondrodysplasia in rat offspring through the Srsf1‐Fn1 axis.

## 3 Discussion

### 3.1 PPE Causes Offspring Chondrodysplasia and Increases Susceptibility to OA in Adulthood

The long‐term and repeated use of prednisone during pregnancy can cause multiple organ dysplasia and various diseases in offspring.^[^ ^9^, ^11^, ^12^, ^13^, ^14^ ^]^ Clinically, mothers often receive a minimum oral dose of prednisone (2.5 mg) daily throughout pregnancy to manage conditions, such as rheumatoid arthritis, asthma, and systemic lupus erythematosus.^[^ ^38^ ^]^ Based on the calculation for a pregnant woman weighing 60 kg, this dose is equivalent to ≈0.257 mg kg^−1^∙d for rats, according to the dose translation between animals and humans.^[^ ^39^ ^]^ Notably, prenatal exposure to glucocorticoids, such as dexamethasone, can cause low birth weights and organ damage including chondrodysplasia.^[^ ^15^ ^]^ Epidemiology also indicates that nearly half of infants born to mothers who took prednisone during pregnancy exhibit low birth weights,^[^ ^9^, ^11^, ^13^ ^]^ which is closely associated with chondrodysplasia and susceptibility to OA in the offspring.^[^ ^40^ ^]^ Therefore, in this study, prednisone was administered intragastrically to pregnant rats at doses of 0.125 and 0.25 mg kg^−1^∙d during GD 0–20 to simulate clinical use throughout pregnancy, which has important clinical significance.

Our results showed that PPE significantly reduced the body length and weight of offspring, the proliferative zone area (round and columnar zone)^[^ ^41^ ^]^ of fetal cartilage and the expression of proliferation‐related protein (Ki‐67), but increased the proliferative resting zone area (hypertrophic zone)^[^ ^41^ ^]^ in fetal rat cartilage. PPE also decreased the cartilage matrix content and the expression of matrix synthesis‐related genes in fetal and adult offspring and decreased the expression of genes related to matrix degradation in utero, whereas these were increased at PW12 and PW28, which is consistent with previous studies of prenatal exposure to another glucocorticoid, dexamethasone.^[^ ^15a^ ^]^ The decrease in the expression of genes related to cartilage matrix degradation may be compensatory, considering the decreased matrix content and synthesis in utero. However, the cartilage quality was already compromised in the PPE group after birth, making it more susceptible to matrix degradation following stimuli. Additionally, after long‐distance running, the PPE combined with the running group showed a more significant decrease in cartilage matrix content and synthesis, and a more pronounced increase in cartilage matrix degradation and OARSI score compared with the running‐only group. Furthermore, typical changes associated with OA, such as fibrosis and cracks, were observed on the cartilage surface of PPE combined with a running group. These findings further support the notion that PPE causes chondrodysplasia and increased susceptibility to OA in adulthood.

### 3.2 circGtdc1 Mediates PPE‐Induced Offspring Chondrodysplasia and Susceptibility to OA in Adulthood

Epigenetics mediate intrauterine organ dysplasia and adult susceptibility to multiple diseases.^[^ ^42^ ^]^ For example, decreased expression of the small non‐coding RNA miR‐98‐3p mediates reduced bone mass in adult offspring induced by prenatal dexamethasone exposure,^[^ ^42^ ^]^ and decreased expression of the long non‐coding RNA H19 mediates adrenal dysplasia and dysfunction in adult offspring induced by prenatal caffeine exposure.^[^ ^43^ ^]^ As non‐coding RNAs, circRNAs have gained increasing attention owing to their roles in disease occurrence, organ development, and intrauterine programming. Various circRNAs, including circSamd4,^[^ ^19^ ^]^ circHomer 1,^[^ ^20^ ^]^ and circPDE4B,^[^ ^24^ ^]^ are associated with skeletal muscle and brain development and OA pathogenesis. However, whether circRNA regulates cartilage development and OA susceptibility in adulthood remains unexplored. In this study, circRNA sequencing revealed 6199 circRNAs in the fetal cartilage, with 4914 and 4813 detected in the control and PPE groups, respectively. Most of these circRNAs were derived from exons with lengths ranging from 0 to 1000 nt, which is similar to previous reports in adult cartilage.^[^ ^24^ ^]^ Furthermore, circGtdc1 was derived from exon 4 of Gtdc1 through back‐splicing. It is produced by the protein‐coding sequence of Gtdc1, which is therefore relatively conserved between rats and humans.^[^ ^24^ ^]^ PPE significantly decreased the expression of circGtdc1 in the cartilage at GD20, PW12, and PW28, but did not change the expression of its linear RNA mGtdc1. Moreover, in vitro, the knockdown of circGtdc1 inhibited chondrocyte proliferation and the expression of genes related to extracellular matrix synthesis, whereas circGtdc1 overexpression had the opposite effect. These results indicate that circGtdc1 (rather than Gtdc1) mediates the chondrodysplasia and OA susceptibility in offspring.

Physiologically, prednisone has no activity and must be converted into prednisolone by 11β‐HSD1 in the liver to exert its effects.^[^ ^9^ ^]^ To further investigate the effects of PPE on cartilage in vitro, we measured the concentrations of prednisone and prednisolone in serum, which ranged from 2.1 to 50.8 and 4.1 to 57.2 nm, respectively. This is consistent with a previous report by Blanford et al. that 51% of prednisolone could cross the placental barrier.^[^ ^10^ ^]^ Therefore, we treated fetal rat chondrocytes with prednisone and prednisolone based on the serum concentrations. We found that prednisone had no significant effect on fetal rat chondrocytes, while prednisolone concentration‐dependently reduced chondrocyte proliferation and the expression of genes related to matrix synthesis. Prednisolone exerts its effects through the GR.^[^ ^26^ ^]^ Further evaluation of the effects of the GR inhibitor RU486 on prednisolone action revealed that RU486 could reverse all the aforementioned effects of prednisolone. This suggests that PPE causes chondrodysplasia through prednisolone (rather than prednisone). Consistent with the tissue results, prednisolone also concentration‐dependently reduced the expression of circGtdc1 in fetal chondrocytes without impacting mGtdc1 expression. In mechanism, prednisolone decreased the expression of eIF4A3, diminishing its binding to the flanking regions of circGtdc1, consequently reducing circGtdc1 expression, which is similar to the change in some circRNA expression such as circRNA‐CRET.^[^ ^44^ ^]^ Additionally, RU486 also inhibited the prednisolone‐induced decrease in circGtdc1 expression. Moreover, circGtdc1 overexpression reversed the inhibitory effects of prednisolone on chondrocyte proliferation, decreased the matrix content, and reduced the expression of genes related to cartilage matrix synthesis. These results indicate that circGtdc1 mediates the chondrodysplasia and susceptibility to OA in offspring resulting from PPE by inhibiting chondrocyte proliferation and extracellular matrix synthesis.

### 3.3 circGtdc1‐Srsf1 Interaction Regulating Downstream Signals Mediates PPE‐Induced Chondrocyte Proliferation and Matrix Synthesis Disorders

Previous studies have identified four main mechanisms through which circRNAs exert their biological functions^[^ ^23^, ^28^ ^]^: influencing gene expression by regulating miRNAs through the ceRNA mechanism^[^ ^23^ ^]^; peptide translation^[^ ^45^ ^]^; regulation of parent gene expression^[^ ^46^ ^]^; and binding with RNA‐binding proteins.^[^ ^28^ ^]^ The mechanism of circRNAs is often associated with their subcellular localization, with nuclear circRNAs primarily associated with protein modification and splicing regulation and cytoplasmic circRNAs associated with ceRNA and molecular scaffolds.^[^ ^47^ ^]^ We found that circGtdc1 was primarily located in the cell nucleus, suggesting its involvement in protein binding and splicing regulation. Through various experimental validations, we confirmed that circGtdc1 regulates Srsf1 protein degradation by affecting the interaction between Srsf1 protein and the E3 ligase BTRC via K48‐linked polyubiquitination rather than via autophagy. Similarly, Wang et al. reported that K48‐ and K63‐linked polyubiquitin chains are the two main types of polyubiquitin linkages in mammalian cells: K48‐linked polyubiquitin usually induces the degradation of substrate proteins while K63‐linked polyubiquitin is correlated with protein stabilization or activation. Moreover, they reported that circRNA‐CREIT can regulate the K48‐linked polyubiquitination of RNA‐activated protein kinase, thereby promoting its degradation.^[^ ^44^ ^]^ The in vitro and in vivo experiments in this study also confirmed that the decrease in Srsf1 protein levels caused by prednisolone and PPE was related to downregulated circGtdc1 expression induced by prednisolone, thus increasing K48‐linked polyubiquitination and the degradation of Srsf1.

Srsf1 belongs to the SR splicing factor protein family, which regulates alternative RNA splicing by initiating spliceosome assembly and activation.^[^ ^36^ ^]^ Alternative RNA splicing allows eukaryotic cells to produce a large number of proteins from a limited number of genes; coordinated alternative splicing networks have important physiological functions in mammalian development.^[^ ^48^ ^]^ Studies have shown that SR proteins play important roles in the development and occurrence of diseases, such as tumors.^[^ ^36^, ^49^ ^]^ The study by Yu et al. demonstrated that Srsf1‐regulated alternative splicing is necessary for dental progenitor cell proliferation and survival.^[^ ^49^ ^]^ The study by Xie et al. also suggested that Srsf1 induces the formation of neointima in blood vessels by promoting the proliferation of vascular smooth muscle cells.^[^ ^50^ ^]^ However, the role of Srsf1 in cartilage development has not been previously reported. In this study, prednisolone and PPE significantly reduced Srsf1 protein expression in chondrocytes and rat cartilage tissues at both GD20 and PW28. Meanwhile, the in vitro knockdown of Srsf1 significantly decreased chondrocyte proliferation, matrix content, and the expression of genes related to matrix synthesis and reversed the increased indicators induced by circGtdc1 overexpression in the presence of prednisolone. These suggest that the circGtdc1‐Srsf1 axis mediates the inhibition of chondrocyte proliferation and matrix synthesis induced by prednisolone in fetal rat chondrocytes.

Fibronectin (encoded by the single Fn1 gene), a multifunctional glycoprotein expressed on the surface of chondrocytes, is a component of the extracellular matrix and also an important molecule in signal transmission between the extracellular matrix and cell surface proteins. Further, it plays an important role in regulating cell adhesion, proliferation, apoptosis, and migration.^[^ ^37^, ^51^ ^]^ Fibronectin is the first protein to appear during embryonic cartilage development and is one of the most important collagens; its expression is essential for cartilage formation.^[^ ^52^ ^]^ To further investigate the downstream mechanisms of Srsf1, we predicted Srsf1‐regulated alternative splicing target genes and performed sequencing following Srsf1 knockdown in fetal rat chondrocytes. The prediction and sequencing results showed that Srsf1 regulated three major signaling pathways (extracellular matrix‐receptor interaction, focal adhesion, and PI3K/AKT) in chondrocytes, with only one common target gene, Fn1. In cartilage, Fn1 is primarily present in alternative splicing forms including EDA^+^, EDB^+^, and EDV^+^ Fn1.^[^ ^51^ ^]^ EDA^+^ and EDB^+^ Fn1 are the predominant splicing variants during cartilage development, and their deficiency or reduced expression leads to abnormal cartilage and skeletal development.^[^ ^52a^ ^]^ Sun et al. demonstrated that increased Srsf1 expression promotes the formation of EDA^+^Fn1 in human and mouse lung fibroblasts.^[^ ^53^ ^]^ Lim et al. also showed that Srsf1 can increase the formation of EDB^+^Fn1 in rats and humans through the TGCATG repeat sequence.^[^ ^54^ ^]^

In this study, the expression of the EDA^+^ and EDB^+^ Fn1 subtypes was reduced in the rat cartilage induced by PPE at GD20 and PW28. Meanwhile, treatment with Srsf1 siRNA or prednisolone also significantly decreased the expression of EDA^+^ and EDB^+^ Fn1 subtypes in chondrocytes, whereas Srsf1 overexpression reversed these effects. EDA^+^Fn1 has been shown to increase the affinity of fibronectin for integrins, thereby affecting PI3K/AKT activity and regulating cell proliferation.^[^ ^51^ ^]^ Additionally, EDA^+^Fn1 has also been shown to promote activation of the TGFβ signaling pathway in cartilage by binding to the latent TGFβ binding protein 1, thereby regulating extracellular matrix formation.^[^ ^55^ ^]^ Therefore, we examined the effects of prednisolone and PPE on Fn1 binding to the integrin receptor and PI3K/AKT and TGFβ pathways. The results showed that prednisolone significantly inhibited Fn1 binding to ITGα5 and the activation of the PI3K/AKT and TGFβ signaling pathways. Furthermore, Srsf1 overexpression reversed the prednisolone‐induced inhibition of Fn1 binding to ITGα5 and activation of the PI3K/AKT and TGFβ signaling pathways, while EDA/B^+^Fn1 knockout abolished these effects of Srsf1. PPE also inhibited the binding of Fn1 to ITGα5 and the activation of the PI3K/AKT and TGFβ pathways in rat cartilage tissue on GD20 and PW28. Collectively, the Srsf1‐induced reduction of EDA/B^+^Fn1 inhibited the activation of the PI3K/AKT and TGFβ signaling pathways, which mediated the decreased chondrocyte proliferation and reduced extracellular matrix synthesis induced by PPE.

### 3.4 circGtdc1‐Srsf1 Mediates PPE‐Induced Offspring Chondrodysplasia and Making circGtdc1 a Potential Interventional Target

Fetal‐derived diseases, such as fetal‐derived OA, exhibit significant challenges to early prevention and treatment owing to their unclear etiology, pathogenic mechanisms, and lack of intervention targets.^[^ ^56^ ^]^ circRNAs, which exhibit good tissue specificity, conservation, and stability, are highly expressed in slow‐dividing cells, such as chondrocytes, making them a favorable choice as intervention targets.^[^ ^47^ ^]^ A study from Zhao et al. demonstrated that circRNA SCAR injected through the tail vein could alleviate high‐fat diet‐induced mouse cirrhosis.^[^ ^28^ ^]^ Meanwhile, Shen et al. showed that intra‐articular injection of AAV‐circPde4b or circSERPINE2 could reverse OA‐like pathological changes in cartilage induced by destabilization of the medial meniscus in mice^[^ ^23^ ^]^ or by anterior cruciate ligament transection in rabbits.^[^ ^24^ ^]^ In this study, intra‐articular injection of AAV‐circGtdc1 to PPE‐induced offspring rats significantly increased cartilage matrix synthesis and content and reduced OARSI scores, thereby improving the poor cartilage quality caused by PPE. Moreover, intra‐articular injection of AAV‐circGtdc1 also significantly reversed the effects of PPE on the expression of total Fn1 and formation of EDA^+^ and EDB^+^ Fn1 subtypes and reduced the binding of Fn1 to integrin receptors. Conversely, intra‐articular injection of AAV‐shRNA(Srsf1) reversed all the effects induced by intra‐articular injection of AAV‐circGtdc1. Therefore, these findings suggest that circGtdc1 overexpression could improve PPE‐induced poor cartilage quality through the Srsf1‐Fn1 axis in vivo and that circGtdc1 could serve as an interventional target for fetal‐derived chondrodysplasia.

## 4 Conclusion

**Figure 8:**
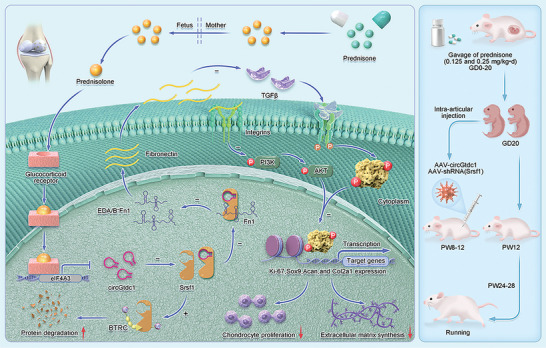
Schematic illustration of PPE‐induced chondrodysplasia and osteoarthritis susceptibility in offspring. Circular RNA Gtdc1 inhibited chondrocyte proliferation and matrix synthesis through Srsf1‐Fn1 signal axis end‐mediated chondrodysplasia and the susceptibility to osteoarthritis in offspring induced by PPE. PPE, prenatal prednisone expression.

As shown in **Figure** **8**, this study demonstrated through both in vitro and in vivo experiments that prenatal exposure to clinical doses of prednisone can dose‐dependently reduce chondrocyte proliferation and extracellular matrix synthesis, leading to chondrodysplasia and increased susceptibility to OA after birth. The mechanism studies suggested that PPE alters the expression profile of circRNAs in fetal rat cartilage, and the decreased expression of circGtdc1 led to reduced binding with Srsf1 protein, thereby facilitating the interaction between Srsf1 and the E3 ligase BTRC to promote degradation of Srsf1 via K48‐linked polyubiquitination, further inhibiting the formation of EDA^+^ and EDB^+^Fn1 isoforms and the activation of the PI3K/AKT and TGFβ signaling pathways. The alteration of the circGtdc1‐Srsf1‐EDA/B^+^Fn1 signaling axis, mediated by prednisolone (rather than prednisone), contributed to the inhibition of chondrocyte proliferation and matrix synthesis, resulting in chondrodysplasia and an increased susceptibility to OA caused by PPE. Intra‐articular injection of AAV‐circGtdc1 ameliorated PPE‐induced chondrodysplasia, which was reversed by the intra‐articular injection of AAV‐shRNA(Srsf1). This indicates that circGtdc1 could represent an interventional target for fetal‐derived OA and that the circGtdc1‐Srsf1 axis mediates the occurrence of fetal‐derived OA induced by PPE. To the best of our knowledge, this is the first study to report the involvement of circRNAs in cartilage development and their potential role in mediating OA occurrence in adulthood, providing a theoretical and experimental basis for studying the etiology, pathogenesis, and early intervention in OA.

## 5 Experimental Section

Detailed materials and methods are included in the supplementary data file.

## Conflict of Interest

The authors declare no conflict of interest.

## Author Contributions

L.L. and Y.H. contributed equally to this work. L.L. performed conceptualization, formal analysis, investigation, data curation, methodology, and visualization, wrote the original draft, and also wrote reviewed, and edited the final manuscript. Y.H. performed conceptualization, formal analysis, investigation, data curation, methodology, and validation. C.M., F.Z., and Q.L. performed investigation, methodology, data curation, and formal analysis. B.L. performed methodology, and project administration, and wrote, reviewed, and edited the final manuscript. H.H. and J.Z. performed formal analysis and methodology. H.W. performed, conceptualization, funding acquisition, and project administration and wrote, reviewed, and edited the final manuscript. L.C. performed conceptualization, funding acquisition, resources, and supervision and wrote, reviewed, and edited the final manuscript.

## Supporting information

Supporting Information

## Data Availability

The data that support the findings of this study are available from the corresponding author upon reasonable request.
