# Supplementary material for: Circular RNA Gtdc1 Protects Against Offspring Osteoarthritis Induced by Prenatal Prednisone Exposure by Regulating SRSF1‐Fn1 Signaling
Source: Adv Sci (Weinh). 2024 Mar 22;11(20):2307442. doi: 10.1002/advs.202307442 (PMC11132075; doi:10.1002/advs.202307442)
Supplement: Supplementary file 1 — Supporting Information [file ADVS-11-2307442-s001.pdf]

ADVANCED SCIENCE
Open Access

Supporting Information

for *Adv. Sci.*, DOI 10.1002/advs.202307442

Circular RNA Gtdc1 Protects Against Offspring Osteoarthritis Induced by Prenatal Prednisone Exposure by Regulating SRSF1-Fn1 Signaling

Liang Liu, Yuntian Hong, Chi Ma, Fan Zhang, Qingxian Li, Bin Li, Hangyuan He, Jiayong Zhu, Hui Wang and Liaobin Chen**

SUPPLEMENTARY MATERIALS AND METHODS

1 Materials and reagents

Prednisone acetate tablets (No. H33021207) were purchased from Xianju Pharma™ (Taizhou, China). Prednisone (CAS. 53-03-2, 98% purity) and prednisolone (CAS. 50-24-8, 98% purity) were purchased from Macklin Biochemical™ (Shanghai, China). Isoflurane was obtained from Antemuye™ (Jinan, Chian). DMEM/F-12 medium and fetal bovine serums (FBS) were purchased from Gibco™ (Grand Island, NY, USA). HiScript III RT SuperMix for qPCR (+gDNA wiper) kit (R323-01), Taq Pro Universal SYBR qPCR Master Mix kit (Q712-02), and 2×Taq Plus Master Mix kit (P211-01) were procured from Vazyme Biotech™ (Nanjing, China). Actinomycin D (AD) (CAS. 50-76-0, HY-17559) and Bafilomycin A1 (BafA1) (CAS. 88899-55-2, HY-100558) were purchased from MedChemExpress™ (Monmouth Junction, NJ, USA). RIPA lysis buffer (P0013B), phenylmethanesulfonyl fluoride (PMSF) solution (ST507), BCA Protein Assay kit (P0010), and MG-132 (S1748-5 mg) were purchased from Beyotime™ (Shanghai, China). Super Signal West Femto Substrate (D046-250 mL) was purchased from Bridgen® (Beijing, China). Ribonuclease R (RNase R) (Cat. RNR07250) was obtained from Biosearch Technologies™ (Petaluma, CA, USA). Primary antibody dilution buffer (G2025), Alcian solution (G1027), DAPI (G1012), Safranin O (G1053-1), and Fast green (G1053-2) were purchased from Servicebio™ (Wuhan, China). Trizol® and Collagenase II was obtained from Invitrogen™ (Carlsbad, CA, USA). Triton® X 100 (CAS. 9002-93-1) was obtained from Biofroxx™ (Einhausen, Hessen, Germany). Mifepristone (RU486) (CAS. 84371-65-3) was procured from Sigma-Aldrich Co., Ltd. (St. Louis, MO, USA). Confocal dishes (BS-15-GJM) and SDS-PAGE Sample loading buffer (5×) (BL502A) were purchased from Biosharp Life sciences™ (Hefei, China). EdU Kit® (MA0425) was purchased from Meilunbio™ (Dalian, China). Collagen type II alpha 1 (Col2a1) (ab34712) for immunohistochemical (IHC) staining, aggrecan (Acan) (ab3778) for IHC and western blotting, SRY (sex-determining region Y)-box9 (Sox9) (ab185230), SMAD family member 2 (Smad2) (ab40855), and goat anti-rabbit IgG H&L (FITC) (ab6717) antibodies were purchased from Abcam™ (Shanghai, China). Glyceraldehyde 3-phosphate dehydrogenase (Gapdh) (AC001), histone H3 (A2348), Col2a1 (A1560) for western blotting and immunofluorescence (IF), matrix metallopeptidase 13 (Mmp13) (A11148), Acan (A12045) for IF, phospho-phosphoinositide 3-kinase (p-PI3K) (AP0854), AKT serine/threonine kinase (AKT) (A11016), phospho-AKT serine/threonine kinase (p-AKT) (AP0140), SMAD family member 3 (Smad3) (A19115), phospho- SMAD family member 2 (p-Smad2) (AP1338), phospho-SMAD family member 3 (p-Smad3) (AP0727), marker of proliferation Ki-67 (Ki67) (A20018), Cy3 Goat Anti-Rabbit IgG (H+L) (AS007), HRP Goat Anti-Rabbit IgG (H+L) (AS014), ABflo® 488-conjugated Goat Anti-Mouse IgG (H+L) (AS037), ABflo® 594-conjugated Goat Anti-Mouse IgG (H+L) (AS054), HRP Goat Anti-Mouse IgG (H+L) (AS003), Mouse Control IgG (AC011), Rabbit pAb Control IgG (AC005), parkin RBR E3 ubiquitin protein ligase (PARK2) (A0968) and beta-transducin repeat containing E3 ubiquitin protein (BTRC) (A21951) antibodies were purchased from Abclonal™ (Wuhan, China). Serine and arginine rich splicing factor 1 (Srsf1) (12929-2-AP), phosphoinositide 3-kinase (PI3K) (67071-1-Ig), transforming growth factor beta (TGFβ) (21898-1-AP), fibronectin (Fn1) (66042-1-Ig), Integrin Alpha-5 (ITGα5) (10569-1-AP), argonaute 2 (AGO2) (67934-1-Ig), and eukaryotic translation initiation factor 4A3 (eIF4A3) (17504-1-AP) antibodies were purchased from Proteintech™ (Wuhan, China). Ring finger protein 125 (RNF125) antibody (DF4024) was obtained from Affinity Biosciences™ (Jiangsu, China). HA Tag antibody was purchased from Servicebio™ (Wuhan, China). Oligonucleotide primers were purchased from TIANYIHUIYUAN™ (Guangzhou, China). DNA loading dye (6×) was obtained from ThermoFisher Scientific™ (Waltham, MA, USA). DNA Marker (MD101) was purchased from TIANGEN® (Beijing, China). Gelview dye was obtained from KERUI Biotechnology™ (Wuhan, China). The remaining materials and agents were of analytical grade.

2 Ethics statement

All animal experiments were conducted in accordance with the National Institutes of Health's Guide for the Care and Use of Laboratory Animals (Revised 1996). Animal-based studies were conducted in Wuhan University Center

for Animal Experiment/ABSL-III Laboratory and performed in line with ethical guidelines/protocols approved by the Institutional Animal Care and Use Committee (IACUC) of Wuhan University Center for Animal Experiment. This study protocol was approved by the Ethics Committee (Approval no.20210060).

3 In vivo assay: animal handling and protocol

Specific pathogen-free Wistar female and male rats (9 weeks old, weighing approximately 220 g, SCXK 2016-0006, 2019-0010, certification number: 110324210100821442 and 110011211103620237) were obtained from the SPF Biotechnology™ (Beijing, China) and Beijing Vital River Laboratory Animal Technology™ (Beijing, China). Rats were raised under standard conditions (12 h light/dark cycle at 22–24 °C and 60% humidity with free access to food and water). After animals were fed adaptively for 1 week, one male and two female rats were caged together overnight. Gestational day (GD) 0 was set following confirmed intercourse through sperm presence within vaginal smears. Pregnant rats randomly were divided into the CON, PPE(L), and PPE (H) group, which received a gavage of solvent (same volume of 0.5% carboxymethylcellulose sodium), 0.125 or 0.25 mg/kg·d prednisone at 8 AM once daily between GD0 and GD20, respectively.

On GD20, part of the pregnant rats were anesthetized with 2% isoflurane and euthanized, followed by harvesting of fetal rats. Offspring ranging from 8 to 14 per litter were included in this experiment, and 12 litters were included per group (n=12). Since primary osteoarthritis (OA) is more common in women, female offspring were used in subsequent studies [1], while the male offspring were used in a separate study. Blood samples were collected from mother and fetal rats for assaying the concentrations of prednisone and prednisolone. Knee joints of the hind limbs were removed from fetuses. The right hind limbs were fixed within 4% paraformaldehyde for histological analysis (n=5), and the left hind limbs were stored at -80 ℃ until further analysis. The remaining pregnant rats were allowed to deliver naturally, and offspring were adjusted to 14 pups per litter on the first day after birth to ensure nutritional balance, and 12 litters were included in each group. Then, the offspring were fed normally to until postnatal week (PW) 12 or 28. A portion of the rats in the from control and PPE(H) groups were included in the long-distance treadmill running experiment from PW24 to PW28 according to the previous reports [2]. All animals were sacrificed at PW12 and PW28, followed by collection of bilateral knee joints from the hind limbs for further analysis.

Finally, 8-week-old offspring rats induced by PPE were included in the *in vivo* intervention experiments. Rats were randomly divided into six groups (n=12): CON, PPE, PPE+AAV-circGtdc1-Vector, PPE+AAV-circGtdc1, PPE+AAV-circGtdc1+AAV-shRNA(NC), and PPE+AAV-circGtdc1+AAV-shRNA(Srsf1) groups (n=12). OBiO Technology™ (Shanghai, China) created and packaged adeno-associated virus (AAV) vectors for circGtdc1 and Srsf1 shRNA. A total of 50 μL (approximately 1×10^{12} vg/mL) of AAV-circGtdc1-Vector, AAV-circGtdc1, or a mixture including AAV-circGtdc1 (25 μL) + AAV-shRNA(NC) (25 μL) or AAV-circGtdc1 (25 μL) + AAV-shRNA(Srsf1) (25 μL) was delivered intra-articularly into the knee joints for 4 weeks [3]. Then, rats were anesthetized with 2% isoflurane and euthanized *via* cervical dislocation, followed by harvesting of right knee joints from the hind limbs. Some samples (n=5) were fixed in 4% paraformaldehyde for histological analysis, and the remaining knee joint samples were stored at -80 ℃ until further analysis. AAV vectors are AAV2/8 serotype and the detailed names of AAVs in this study are as follows: AAV-circGtdc1-Vector, pAAV-CMV-MCS-GdGreen-WPRE; AAV-circGtdc1, pAAV-CMV-S-circGtdc1-EF1-GdGreen-WPRE; AAV-shRNA(NC), pAAV-U6-sh(NC)-CMV-mScarlet-WPRE; AAV-shRNA(Srsf1), pAAV-U6-sh(Srsf1)-CMV-mScarlet-WPRE.

4 Serum prednisone and prednisolone concentration measurements

After serum and standard samples (for calibration curve) were prepared, prednisone and prednisolone concentrations were assayed using liquid chromatography-tandem mass spectrometry (LC-MS/MS) [4]. Briefly, after an appropriate internal standard and extraction solvent (1:1 methanol-to-water and 0.1% formic acid) was

added to the serum sample, vortexed for 30 s, and centrifuged them at 12000 rpm for 10 min. The supernatant was transferred to a new EP tube and dried using a vacuum concentrator. Then, the dried extract was reconstituted in a suitable volume of mobile phase and assayed by LC-MS/MS according to the instrument manufacturer's instructions. Finally, the prednisone and prednisolone concentration was quantified based on the calibration curve.

5 Alcian blue and Alizarin red staining

After the pregnant rats were sacrificed on GD20, the fetuses were removed and fixed in 95% ethanol for 1-2 weeks. Then, the skin and internal organs were removed with eye tweezers, and samples were soaked in 100% acetone for 2-3 d. Next, samples were stained with Alcian blue and Alizarin red, respectively. The soft tissues were then cleared, and the contrast of bones was enhanced using 1% KOH treatment for 1-2 d. Finally, after transparent in 20% glycerol for 1 week, samples were transferred to 50% glycerol for photography and preservation. The cartilage was stained blue, and the ossification center was stained red [5].

6 Safranin O-fast green staining and Histological scoring

After fixation with 4% paraformaldehyde for 7 d, samples were decalcified in 20% EDTA (pH 7.4), with the decalcification solution was replaced every three days until the tissue was sufficiently softened. The softened samples were dehydrated prior to paraffin embedding. Safranin O-fast green staining was performed on 5 μm-thick sagittal slices using a previously described method [6]. Briefly, the sections were deparaffinized in water (the specimens were sequentially placed in xylene I−xylene II−anhydrous ethanol I−anhydrous ethanol II−90% alcohol−85% alcohol−75% alcohol and then washed with water) and stained with Fast green (G1053-2, Servicebio™, Wuhan, China) for 5 min, followed by Safranin O (G1053-1, Servicebio™, Wuhan, China) for 5 s before rapid dehydration with absolute ethanol. The sections were then sealed with neutral gum and cleared with xylene for 5 min before being scanned with a digital slice-scanner (Aperio® CS2, Leica™, Germany). The post-staining images were analyzed by the mean optical density (MOD) of Safranin O staining (red). In fetal cartilage (GD20), images (40×) (n=5) containing whole cartilage tissue were used to calculate the MODs, while six different visual fields near the surface of the cartilage in adult rats (PW12 and 28) for each sample (n=5) were used to calculate the MODs through ImageJ® software (v.1.52q, National Institutes of Health, Bethesda, MD, USA). Histological scoring used Osteoarthritis Research Society International (OARSI) scoring system by Pritzker [7]. Grade 1.0 represented intact chondrocytes; Grade 1.5 indicated cell death by apoptosis or necrosis; Grade 2.0 corresponded to the surface discontinuity consists of fibrillation; Grade 2.5 signified abrasion of the surface with loss of a portion of the superficial cartilage zone; Grade 3.0 indicated simple fissures or clefts that penetrating the mid-zone; Grade 3.5 represented fissures that extended to become branched or complex; Grade 4.0: indicated loss of the superficial zone only (erosion); Grade 4.5 signified absence of the mid-zone; Grade 5.0 denoted the presence of a bone surface consisting of intact calcified cartilage or sclerotic bone (denudation); Grade 5.5 indicated the presence of reparative fibrocartilaginous tissue or new bone formation; Grade 6.0 corresponded to deformation of the joint geometry at the joint margins; Grade 6.5 signified deformation changes in both the joint margins and force bearing areas. OA staging was based on the horizontal extent of the involved cartilage surface, with Stage 1 indicating less than 10% involvement, Stage 2 indicating 10-25% involvement, Stage 3 indicating 25-50% involvement, and Stage 4 indicating more than 50% involvement. OARSI score = Grade score × Stage score. The final scores were determined independently by two researchers.

7 Immunohistochemical (IHC) assay

After decalcification and embedding of samples, 5 μm sagittal-slices were prepared for IHC staining as previously described [6]. The specimens were deparaffinized in water and antigen retrieval was performed by boiling in sodium citrate buffer. After blocking in BSA for 0.5-1 h, the sections were incubated overnight at 4°C with primary antibodies (1:200 for Acan, Col2a1, Ki-67, Srsf1, and 1:100 for Mmp13) in a humidified chamber.

On the second day, biotinylated secondary antibody was added for 30 min, followed by an avidin-biotinylated horse radish peroxidase complex. Finally, the DAB substrate was used for coloration. The images were scanned using a digital slice-scanner (Aperio® CS2, Leica™, Germany). The staining intensities were determined by positive cells (Ki-67 and Srsf1) or MODs (Col2a1, Acan, and Mmp13) of six different visual fields for each sample (n=5). Post-staining images were analyzed through ImageJ® software (v.1.52q, National Institutes of Health, Bethesda, MD, USA). After converting into 8-bit format, the images were calibrated with uncalibrated OD mode, and MODs of the default thresholds (selected by ImageJ® software) were then calculated.

8 Immunofluorescence (IF)

For IF tissue staining, 5 μm sagittal-slices were first prepared. After baking at 60 °C for 2–8 h, the sections were deparaffinized to water and antigen retrieval was performed by boiling in sodium citric acid buffer for 15 min. After cooling, 0.5% Triton® X-100 was added for 15 min to broken the cell membrane, followed by being blocked for 1 h with 10% BSA in TBST at room temperature for 2 h and incubated overnight with primary antibodies (1:200 for Col2a11 and 1:100 for Acan) at 4 °C. On the second day, a Cy3-conjugated secondary antibody (1:200) was added for 1 h, and the nucleus was stained with DAPI for 15 min at room temperature after samples were rinsed with TBST. Similarly [6], for the cellular IF, after samples were treated in confocal dished, cells were fixed with 4% paraformaldehyde for 15 min, and cell membrane was ruptured by 0.5% Triton® X-100 30 min at room temperature. After rinsing with PBS thrice, the samples were blocked with BSA for 1 h, and were then incubated overnight at 4°C with primary antibody dilution (1:200 for Col2a1, Srsf1, and Fn1; 1:100 for Acan, 1:250 for Sox9, and 1:50 for ITGα5). After rinsing with TBST for 3 times, the cells were incubated with fluorescent secondary antibody in dark for 1 hour. Finally, the samples were stained with DAPI at room temperature for 5 min before rinsing with PBS. IF staining was observed and photographed under a confocal microscope (TCS-SP8-STED®, Leica™, Germany). ImageJ® was used to assay the mean gray values of 4-6 different visual fields for each sample (n=3 or 6 for cellular or tissue IF, respectively). Fiji® software (v.12.9.0/1.53t, National Institutes of Health, Bethesda, MD, USA) was used for protein colocalization analysis by calculating Pearson's correlation coefficients.

9 In vivo Bioluminescence Imaging

After knee joints were harvested and the soft tissue was removed, samples were photographed using a small animal *in vivo* imaging system (Xtreme B1, Bruker™, Germany) under the following parameters: Modality: Fluorescence; Setting: Current Session; Source: Multi-wavelength; Apply Reference File: None; Excitation and Emission: according to the fluorescent label of plasmid (the excitation and emission wavelength of mScarlet is 569/594; GdGreen is 469/506); Exposure Time: 2.00 s; FOV: 10 cm; fStop:2; Focal Plane: 1.1 mm. Other parameters were set to default settings. Bruker MI SE (Bruker™, Germany) software was used for data analysis.

10 RNA extraction & real-time quantitative polymerase chain reaction (RT-qPCR)

Total RNA was extracted from articular cartilage tissues/chondrocytes using the TRIzol reagent, following established protocols [6]. Briefly, samples were mixed with 1 mL of TRIzol and 200 μL chloroform in 1.5 mL EP tubes, then cooled on ice for 10 min after thorough mixing. After centrifugation at 12000 g for 15 min, the top layer (400 μL) was transferred to a new EP tube, mixed with 400 μL isopropanol, and left at room temperature for 10 min. Total RNA was obtained after centrifugation at 12000 g for 10 min, washed twice with 1 mL 75% precooled ethanol, and dissolved in RNA enzyme water. The concentration and purity were analyzed using a NanoDrop 2000 micronucleic acid analyzer (ThemoFisher Scientific™, USA), ensuring an A260/A280 ratio of 1.8–2.0 for all samples. Then, 1 μg total RNA was used to synthesize cDNA using a HiScript III RT SuperMix for qPCR (+gDNA wiper) Kit according to the protocol, and cDNA was amplified Taq Pro Universal SYBR qPCR Master Mix kits using the StepOnePlus™ Real-Time PCR System (AppliedBiosystems by ThermoFisher Scientific™, USA) according to the manufacturer's protocol. The primer sequences are shown in Table S2. The real-time PCR

conditions were as follows: 95°C/30 s, 95°C/10 s and 60°C/30 s (40 cycles). The relative mRNA expression was calculated against Gapdh according to the formula $Y=2^{-\triangle\triangle Ct}$. Briefly, △Ct is equal to the difference in Ct (target) and Ct (reference gene); △△Ct was obtained by subtracting the △Ct of each sample from the average of △Ct of the control group. Finally, the $2^{-\triangle\triangle Ct}$ was calculated to obtain the relative expression of the target gene in each sample [8].

11 RNA sequencing

Fetal cartilage tissue (n=3) was used for circular RNA sequencing (circRNA seq). Chondrocyte samples (n=3) from the control group and Srsf1 siRNA treated group were used for mRNA sequencing. Total RNA was extracted using the TRIzol reagent, and RNA purity and concentration were confirmed using the NanoPhotometer® spectrophotometer (IMPLEN™, CA, USA) and Qubit® RNA Assay Kit in Qubit® 2.0 Fluorometer (Life Technologies™, CA, USA). RNA integrity was assessed using the RNA Nano 6000 Assay Kit of the Bioanalyzer 2100 system (Agilent Technologies™, CA, USA). For circRNA seq, total RNAs were digested with RNase R to enrich circular RNA. Sequencing libraries were generated using NEBNext® UltraTM RNA Library Prep Kit for Illumina® (NEB, USA) following the manufacturer's recommendations. The libraries were purified (AMPure XP system) and library quality was assessed on the Agilent Bioanalyzer 2100 system. Thereafter, sequencing was performed on an Illumina Hiseq platform and 125 bp/150 bp paired-end reads were generated. After raw data was subjected to quality control (removing adapter reads, reads with N > 0.002, and reads with low-quality bases > 50% of the read length), clean reads were aligned to the reference genome using STAR (v2.5.1b). Find_circ [9] and CIRI [10] software were used to identify circular RNAs. Differentially expressed circRNAs with statistical significance between two groups were identified by fold change cutoff or through Volcano Plot filtering. For cellular mRNA seq, sequencing was performed on an Illumina Novaseq platform and 150 bp paired-end reads were generated [11]. Raw reads were first processed through in-house Perl scripts to obtain clean data. At the same time, Q20, Q30, and GC content of the clean data were calculated. All downstream analyses were based on clean data. The clean data with high quality were aligned with the reference genome using Hisat2 V2.0.5. Differential expression analysis was performed using the DESeq2 R package (1.20.0). Genes with an adjusted $P \leq 0.05$ and absolute fold change ≥ 2 were considered differentially expressed genes (DEGs). Gene Ontology (GO) and Kyoto Encyclopedia of Genes and Genomes (KEGG) enrichment analysis of DEGs was conducted using the clusterProfiler R package.

12 Cellular culturing

Fetal primary chondrocytes were isolated from the femurs of newborn Wistar rats with 0.2% Collagenase II in DMEM/F-12 medium [12]. After being isolated and cut into pieces in a clean bench, cartilage tissue digested with 0.2% collagenase II in 5% CO_2 at 37 °C for 8–12 h. Then, chondrocytes were obtain after centrifugation at 500 g for 5 min, which were identified by observing their morphology (spindle-shaped, round or polygonal) and expression of chondrocyte-specific proteins (i.e., Acan and Col2a1) [13]. The cells were plated in DMEM/F-12 medium with 10% FBS,100 μg/mL streptomycin, and 100 U/mL penicillin. After two generations, cells were cultured in a six-well plate and treated with different concentrations of prednisone (10, 50, and 250 μM) or prednisolone (10, 50, and 250 μM) for 48 h with or without RU486 (2.5 μM) when the cells reached approximately 80% confluence according to the experiment design. At least three biological and two technical replicates were set for each assay.

13 Cellular Safranin O & Alcian blue staining

Chondrocytes cultured in six-well plates were rinsed thrice with PBS and fixed with 4% paraformaldehyde for 15 min. Subsequently, the samples were stained with Safranin O (n=6) or Alcian blue (n=5) at room temperature for 5 min, followed by three rinses with PBS, and the staining were observed and photographed by Nikon light microscope (Eclipse Ci-L, Nikon™, Japan). Finally, the quantitative analysis was determined by MOD in six

different visual fields for each sample through ImageJ.

14 5-Ethynyl-2'-deoxyuridine (EdU)

Chondrocytes were cultured in six-well plates and treated according to the experimental design. EdU Kit® (MA0425, Meilunbio™, China) was used to assay the chondrocyte proliferation according to the manufacturer's protocol. After adding preheated EdU (10 μM) at 37 °C to the six-well plates, samples were incubated for 2 h at the same temperature, followed by fixation with 4% paraformaldehyde for 15 min after washing twice with PBS. Subsequently, 0.5% Triton X-100 was added for 10 min at room temperature, and cells were incubated in Click solution (composed of 430 μL Click Reaction Buffer, 20 μl $CuSO_4$, 1 μL 555-Azide, and 50 μL Click-iT Additive Solution for each sample) for 30 min at room temperature in dark after three rinses with PBS. Finally, samples were photographed using a fluorescence microscope (EVOS fl auto, ThermoFisher Scientific™, USA) and the quantitative analysis of six different visual fields for each sample was evaluated using ImageJ software.

15 RNase R & actinomycin D (AD) assays

For the RNase R assay, 2 μg of total RNA (n=3) was incubated for 15 min in a 37 °C water bath with 3 U/μg RNase R (Cat. RNR07250, Biosearch Technologies™, USA). Subsequently, the expression levels of circGtdc1 and linear mRNA Gtdc1 (mGtdc1) were assessed by RT-qPCR. For the AD assay, primary fetal rat chondrocytes (n=3) were cultured in a six-well plate and treated with 2 μg/ml AD (CAS. 50-76-0, HY-17559, MedChemExpress™, USA) for 4, 8, 12, or 24 h. Finally, cells were collected and RT-qPCR was used to confirm the expression levels of circGtdc1 and mGtdc1.

16 Western blotting analysis

Western blotting was performed as described previously [12]. Briefly, cells were rinsed with ice-cold PBS and lysed in a RIPA lysis solution containing 1 mM PMSF on ice for 30 min. Samples were collected and centrifugated at 12000 g and 4 °C for 10 min. The supernatants were transferred to a new EP tube to obtain the total protein. The protein concentration was determined by BCA Protein Assay kit (P0010, Beyotime™, Shanghai, China), and all samples adjusted to the same concentration. Samples were boiled for 10 min after adding SDS-PAGE sample loading buffer (5×) (BL502A, Biosharp Life sciences™, China) and mixed. Next, equal amounts of samples (30 μg per lane) were resolved by 10% SDS-PAGE gel at 120 voltage and transferred onto PVDF membranes (Millipore, MA, USA) using 300 mA current. The membranes were blocked in in TBST solution supplemented with 5% non-fat milk for 1 h at room temperature, followed by overnight incubation at 4 °C with primary antibodies for Sox9 (1:1000 dilution), Acan (1:200 dilution), Col2a1 (1:500 dilution), Srsf1 (1:1000 dilution), HA (1:500 dilution), PI3K (1:1000 dilution), p-PI3K (1:500 dilution), AKT (1:500 dilution), p-AKT (1:500 dilution), TGFβ (1:1000 dilution), Smad2 (1:2000 dilution), Smad3 (1:2000 dilution), p-Smad2 (1:1000 dilution), p-Smad3 (1:500 dilution), Fn1 (1:2000 dilution), PARK2 (1:500 dilution), BTRC (1:500 dilution), RNF125 (1:500 dilution), Gapdh (1:10000 dilution), and H3 (1:500 dilution). After three rinses with TBST, the membranes were incubated with HRP-linked secondary antibodies (1:5000 dilution) at room temperature for 1.5 h. Finally, immunoreactive bands were detected using Super Signal West Femto Substrate (D046-250 mL, Bridgen®, China) after three rinses with TBST. Densitometric analysis was performed using Image J software.

17 RNA pull-down & Silver staining & Mass Spectrometry (MS)

The RNA-binding protein assay was performed using an RNA pulldown Kit (Bes5102, BersinBio™, China) [14]. Briefly, biotin-labeled specific circGtdc1 and antisense probes, designed and synthesized by RiboBio™ (Zhouguang, China), were incubated with streptavidin magnetic beads at 25 °C for 30 min. After removing nucleic acid *via* addition of DNase and agarose beads according to the kit instruction, total protein samples from fetal rat chondrocytes were mixed with the above complex of magnetic beads and probes and rotated at 25 °C for 2 h. Finally,

the protein samples were obtained by removing the supernatant and washing four times on a magnetic stand (HY-K0200, MedChemExpress™, USA). The retrieved protein samples above were used for silver staining, western blotting, and MS assays [14].

The silver staining assay was performed using a Fast Silver Stain Kit (P0017S, Beyotime™, China). In brief, after performing SDS-PAGE gel electrophoresis of the protein samples (10 µL per lane), the gels were fixed with 100 mL of fixation fluid containing 50 mL of ethanol, 10 mL of acetic acid, and 40 mL of double distilled water (ddH$_2$O). Thereafter, the gels were rinsed with 30% ethanol and ddH$_2$O, separately, and treated with 100 mL of silver stain sensitizer for 2 min on the shaker at 60–70 rpm. Finally, after rinsing with ddH$_2$O twice, the gels were stained with 1% silver solution in ddH$_2$O and photographed with a gel scanner (MicroTek™, China).

The protein MS assay was performed as described previously [15]. Briefly, the retrieved protein samples were dried and incubated with 10 mM dithiothreitol and 55 mM iodoacetamide successively. After drying and washing with NH$_4$HCO$_3$, 50% acetonitrile, and 100% acetonitrile successively, the samples were digested with trypsin at 37 °C overnight, and the polypeptides were extracted by adding an acetonitrile, water, and formic acid (FA) mixture. Subsequently, the concentrated and dried peptides and Ziptip C18 column were desalted and dried, and 1–2 µg of samples in 0.1% FA were analyzed through a 1.9 µm C18 column (75 µm × 20 cm) with the MS platform of Orbitrap Fusion Lumos (ThermoFisher Scientific™, USA) (HPLC liquid phase system: EASY-nLC 1000 (ThermoFisher Scientific™, USA); Flow rate: 200 nL/min; detection: DDA model, Full scan resolution: 60,000(FWHM); m/z: 350-1600; HCD model: 30%). Finally, raw data were analyzed by Proteome Discoverer 2.4 software (Sequent HT, ThermoFisher Scientific™, USA) to obtain protein data, and the proteins identified above were annotated by GO and KEGG to understand the functional properties of different proteins.

18 Cellular silencing & overexpression of genes

The siRNA targeting the junction region of circGtdc1 sequence and Srsf1 overexpression plasmid were designed and synthesized by RiboBio™ (Guangzhou, China). eIF4A3 and BTRC overexpression plasmids were designed and synthesized by GenePharma™ (Suzhou, China). siRNAs of eIF4A3, BTRC, Srsf1 and EDA/B$^+$Fn1 were designed and synthesized by Tsingke Biotech™ (Beijing, China). The pLV-ciR-Gtdc1 overexpression plasmid was constructed by cloning the cDNA of circGtdc1 to pLCDH-ciR vector (Geneseed™, Guangzhou, China). Primary fetal rat chondrocytes were cultured in six-well plate in complete DMEM/F-12 medium and grown to 70% confluence in a monolayer. The transfection of siRNAs or plasmids was performed with a Lipofectamine 3000™ Transfection Kit (Lot. 2383585, Invitrogen™, USA) according to the instruction. 5 µL of siRNA (2 µg plasmid+5 µL P3000™) +125 µL of Opti-MEM™ media (31985-070, Gibco™, USA) and 5 µL of Lipofectamine 3000+125 µL of Opti-MEM™ medium were mixed separately in two EP tubes. After incubation for 5 min, the samples of above two tube were mixed gently and incubated for 15 min at room temperature. The mixture was added to the six-well plate, and the culture medium was supplemented to a final volume of 2 mL/well for an additional culture for 72 h. Finally, samples were collected for further analysis according to the experimental design.

19 Nuclear and cytoplasmic RNA & genomic DNA (gDNA) extraction

Nuclear and cytoplasmic RNAs were extracted from chondrocytes using a Cytoplasmic & Nuclear RNA Purification Kit (Cat. NGB-21000, Norgen Biotek™, Canada). In brief, chondrocytes were collected and lysed by lysis buffer on ice for 15 min according to the kit protocol. After centrifugation at 14000 g for 10 min, the supernatant containing cytoplasmic RNA and the pellet containing the nuclear RNA were transferred to new EP tubes. Subsequently, binding cytoplasmic and nuclear RNA to the column and column wash were performed following the instruction. Finally, after placing the column into a fresh 1.7 mL Elution tube, cytoplasmic and nuclear RNA was obtained by adding 50 µL of Elution Buffer and centrifuging for 2 min, followed by 1 min at 14000 g at 4 °C. The

isolated nuclear and cytoplasmic RNAs were analyzed by RT-qPCR.

The gDNA of chondrocytes was extracted using a TIANamp Genomic DNA Kit (DP304, TIANGEN BIOTECH®, China). All reagents below were provided by the kit. Briefly, after chondrocytes were collected and centrifuged at 11200 g for 1 min, the cells were resuspended with GA buffer. Subsequently, protease K and GB buffer were added to the samples and placed at 70 °C for 10 min, followed by addition of 200 µL of ethanol. After mixing, the samples were transferred to a CB3 column and washed with 500 µL of GD and 600 µL of PW buffer (twice), followed by centrifugation at 13400 g for 30 s. Finally, gDNA was obtained by adding TE buffer for 2–5 min before centrifugation at 13400 for 30 s. gDNA was used to analyze the expression of circGtdc1 and mGtdc1 by agarose gel electrophoresis analysis.

20 Immunoprecipitation (IP)

After the chondrocytes were treated according to the experimental design and rinsed with ice-cold PBS thrice, IP lysis buffer (P0013B, Beyotime™, China) containing a protease inhibitor (1 mM PMSF) was added, and the cells were lysed at 4 °C for 30 min. The samples were then collected and transferred to an enzyme-free EP tube. After centrifugation at 4 °C and 14000 g for 15 min, 100 µL of supernatant was transferred to a new EP tube as the Input group. The remaining supernatants were divided evenly into two groups and were added 1 µg of Srsf1 or IgG antibodies and incubated overnight at 4 °C. Protein antibody complexes were then mixed with protein agarose beads and incubated at room temperature for 3–5 h, and the precipitate was obtained after centrifugation at 4 °C and 14000 g for 15 min. Finally, after the complexes were washed with ice-cold IP lysis buffer, the precipitated proteins were eluted and denatured in 5× SDS-PAGE sample loading buffer, followed by western blotting analysis.

21 Ubiquitination (Ub) assay

Chondrocytes were transfected with specific Ub plasmids (K6, K11, K27, K29, K33, K48, and K63) combined with siRNA for circGtdc1 or a negative control for 72 h. Then, cells were collected after treating with 20 µM MG132 (HY-13259, MedChemExpress™, USA) for an additional 6 h. Finally, the samples were used for IP and western blotting assay to analyze the ubiquitination of Srsf1 protein.

22 Agarose gel electrophoresis

circGtd1 and mGtdc1 were amplified from gDNA and cDNA using PCR with the 2×Taq Plus Master Mix kit (P211-01, Vazyme Biotech™, China). Then, the PCR products (mixed with DNA loading dye) were electrophoresed in 1.2% agarose containing Gelview dye (1 µL/10 mL agarose gel) under 150 voltage and stopped at 2 cm from the edge of the gel. Finally, the gels were observed and photographed using a gel imaging system (JY04S-3C, Beijing JUNYI Electrophoresis Co., Ltd., China)

23 Flow cytometry

Chondrocyte apoptosis was assayed using an Annexin V - FITC/PI double staining cell detection kit (BB-4101-50T, BestBio™, China) [16]. Briefly, primary fetal rat chondrocytes (n=3) were collected and rinsed with PBS thrice by centrifugation (4 °C, 500 g for 5 min) after treated by different concentrations of prednisone (0, 10, 50, and 250 µM) or prednisolone (0 and 250 µM) for 48 h. Subsequently, the cells were resuspended in 400 µL 1× Annexin V binding solution. 5 µL of Annexin V - FITC staining solution was then added and incubated for 15 min at 4 °C in darkness, followed by addition of 5 µL of PI staining solution and incubation for 5 min at 4 °C in the dark. Finally, samples were analyzed immediately using flow cytometry (CytoFLEX S, Beckman Coulter™, USA).

24 RNA immunoprecipitation (RIP) assay

The RIP assay was used to detect the binding between circGtdc1 and AGO2 or Srf1 protein by RIP Kit (Cat. No.

P0102, Geneseed™, China) according to the manufacturer's instructions. Briefly, protein samples were extracted from the chondrocytes and mixed with protease and RNase inhibitors. After centrifugation at 4 °C and 14000 g for 10 min, 100 μL of supernatant was transferred to a new EP tube as the input. The remaining samples were incubated with protein A+G beads conjugated with AGO2/Srsf1 or IgG antibodies at 4 °C and 10 rpm overnight. After washing, the RNA was extracted from the above magnetic bead complex and Input samples. Finally, RT-qPCR was used to determine the expression of circGtdc1.

25 Statistical analysis

SPSS 20® (SPSS Science Inc™, USA) and Prism 8® (Graph Pad Software™, USA) software were employed for all analyses and graphical representations. Quantitative data were expressed as the mean ± standard error of the mean (S.E.M.); qualitative data were presented as the median with an interquartile range. Comparisons between two groups were performed by two-tailed unpaired Student's t-test. One-way analysis of variance (ANOVA) was used for more than two groups, followed by *post hoc* multiple comparisons when homogeneity of variance and normal distribution were observed. For quantitative data with no homogeneity of variance or non-parametric distribution and qualitative data, the Kruskal-Wallis H test was used prior to pairwise comparison with the Nemenyi test. $P < 0.05$ was considered statistically significant.

SUPPLEMENTARY TABLES AND FIGURES

Table S1. The prednisone and prednisolone concentration in rat serum induced by prenatal prednisone exposure

Groups (mg/kg·d)	Prednisone (nmol/L)	Prednisolone (nmol/L)
Mother (0.125)	27.5±10.9 (15.3−50.8)	22.9±10.1 (6.0−35.5)
Fetuses (0.125)	7.5±3.7 (2.1−11.9)	11.5±3.4 (6.2−14.8)
Mother (0.25)	23.5±7.3 (15.1−32.6)	20.1±16.8 (3.5−57.7)
Fetuses (0.25)	9.9±6.5 (3.5−21.1)	21.9±17.9 (4.1−57.2)

Table S2. Primers used for RT-qPCR

Genes（Rat)	Forward primer (5'→3')	Reverse primer(5'→3')	Product(bp)
Gapdh	GCAAGTTCAACGGCACAG	GCCAGTAGACTCCACGACA	140
Mmp3	CCAAGAGAGAGTGTGGATTCTG	TGAGCAGCAACCAGGAATAG	278
Mmp13	CCCTGATGTTTCCCATCTATAC	GTCACACTTCTCTGGTGTTT	143
Adamts5	GCAACAAAGTGGGACTACA	GAGAGAATGCATCCCTTAGC	148
Sox9	GTCGGTGAAGAATGGGCAAG	GCTTGCACGTCTGTTTTGGG	268
Col2a1	CTGGTGGAGCAGCAAGAGC	GTGGACAGTAGACGGAGGAAAG	144
circRab31	GGAGACAAGTGCGAAGAATGC	TCGCCATCACGATGTTCTCTG	198
mGtdc1	GTCTTGCACTGGGGCTACTT	ACAGCCACAGTACACAGCTT	132
circMap4_0002	CATGGAGTGGAGGGGAATAACA	TCCAGCGCAGCCATAAAGT	136
circRabep1_0025	GCATCCCTCCTCTCAAGTGTC	ACAGCAAGTCTGCATCTAAGGAA	160
circIck_0006	CCGATCAAGACCTCCATACACA	GTCGGGACACTCATCTCAGG	200

circIntergenic_001529	AGACTCCTAACTCCTCCCCCT	ATGTATCTGTTGGGGCTTTGC	200
circArhgap10_0018	TTCGAGTTCGTGGAGCCGAT	TCTCCAAGGGCTTAATCAGGGT	163
circDcaf6_0022	CAAGGTGGTTTGAAGAAGCAAGT	TGCTCTCCATCTCGTTCTCGT	112
circEhmt1_0004	GCAAGACCATGCCGAAGTCC	AGGAGTCTCTCCTGCTTGCTT	155
Srsf1	GGAAGCTGGCAGGACTTAAA	TACGGCTTCTGCTACGACTA	256
CaMK2δ	GGGTGCCATCTTGACAACTA	ATCTGTGAAAGTCCATCCCTTC	287
Creb	AGCCGGGTACTACCATTCTA	TTGCTGCTTCCCTGTTCTT	246
Rac1	GTATCCTGAAGTACGACACCAC	ACAGAGAACGGCTCGGATA	244
EDA^{+}Fn1	GACTGTGTACTCAGAACCCG	ACAGGGTGACCTACTCAAGC	115
EDB^{+}Fn1	CCGCCATTAATGAGAGTGAT	AGTTAGTTGCGGCAGGAGAAG	129
Fn1	CCAGGCACTGACTACAAGAT	CATGATACCAGCAAGGACTT	145
eIF4A3	TGATCCGCCGTAGAAGTTTAAG	CTCATCACGCTTCACCAAGA	227
BTRC	CTCTCAGCAAGCTATGAGAAGG	GGCGTCCAAGTATGACAGAAT	231

RT-qPCR, real-time quantitative PCR; Gapdh, glyceraldehyde 3-phosphate dehydrogenase; Mmp3, matrix metallopeptidase 3; Mmp13, matrix metallopeptidase 13; Adamts5, ADAM metallopeptidase with thrombospondin type 1 motif 5; Sox9, SRY (sex-determining region Y)-box9; Col2a1, collagen type II alpha 1; Acan, aggrecan; Gtdc1, glycosyltransferase like domain containing 1; Rab31, ras-related protein rab-31; Mlip, muscular lmna interacting protein; Rabep1, Rab GTPase-binding effector protein 1;Ick, serine/threonine-protein kinase ICK; Arhgap10, Rho GTPase activating protein 10; Dcaf6, DDB1 and CUL4 associated factor 6; Ehmt1, euchromatic histone lysine methyltransferase 1; Srsf1, serine and arginine rich splicing factor 1; CaMK2δ, Calcium/Calmodulin-dependent protein kinase (CaM Kinase) II delta; Creb, CAMP responsive element binding protein 1; Rac1, Rac family small GTPase 1; Fn1, fibronectin 1; eIF4A3, eukaryotic translation initiation factor 4A3; BTRC, beta-transducin repeat containing E3 ubiquitin protein.

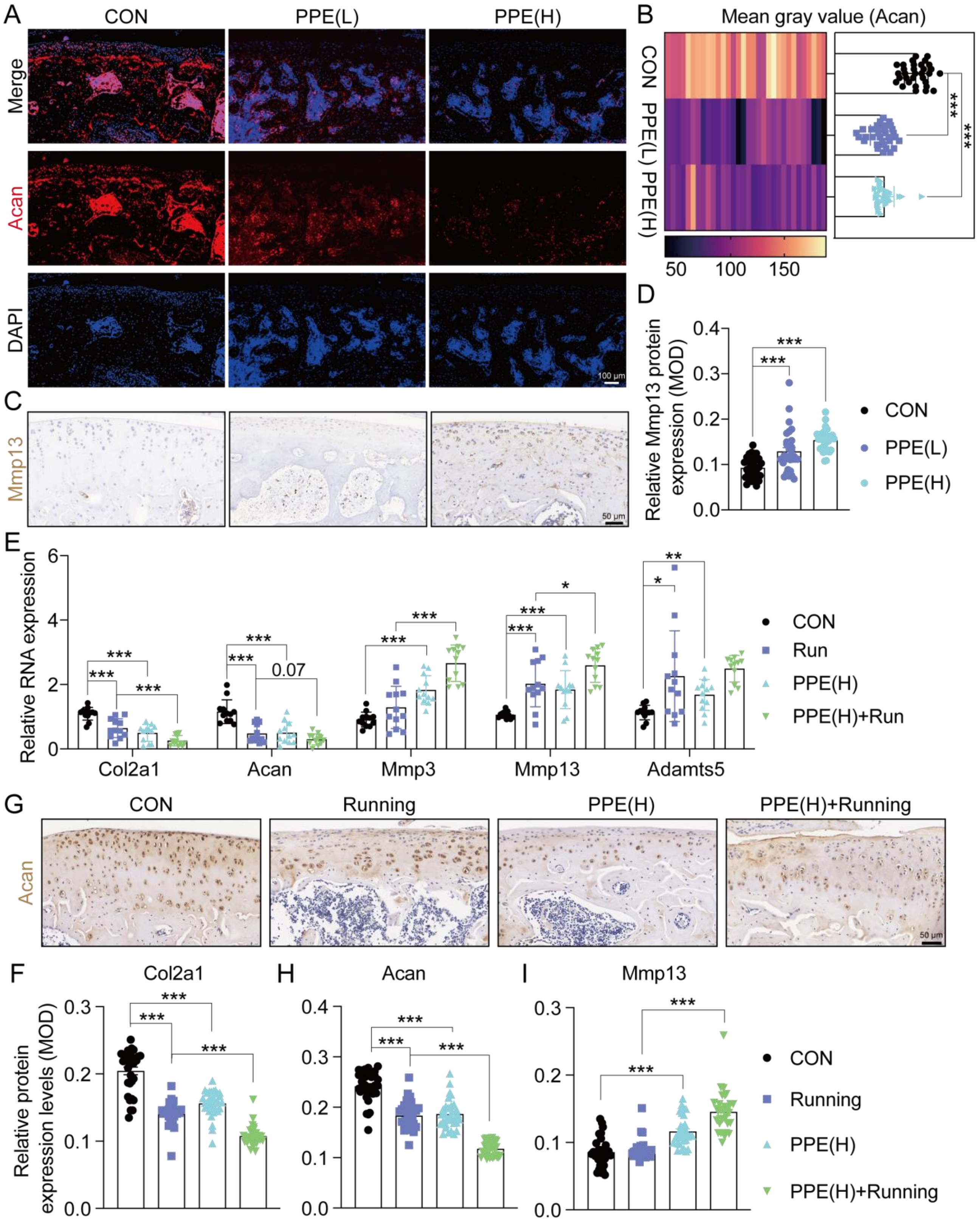

Fig. S1. Influences of PPE on cartilage quality and susceptibility to OA of adult rats. (A, B) The protein expression and quantification of Acan were assayed by immunofluorescence, n=5×6; (C) Immunohistochemical staining was used to detect the level of Mmp13; (D) The quantification (MOD) of Mmp13 protein level by immunohistochemical staining, n=5×6; (E) RT-qPCR was used to detect the mRNA expression of Col2a1, Acan, Mmp3, Mmp13, and Adamts5 in rat cartilage at PW28, n=12; (F) The quantification (MOD) of Col2a1 protein level, n=5×6; (G) Immunohistochemical staining was used to assay the Acan protein level in rat cartilage at PW28; (H, I) The quantification (MOD) of Acan and Mmp13 protein levels in rat cartilage at PW28, n=5×6. Values are expressed as

the means ± S.E.M. $^{*}P < 0.05$, $^{**}P < 0.01$, $^{***}P < 0.001$ *vs.* corresponding control. PPE, prenatal prednisone expression; CON, control; Acan, aggrecan; Mmp13, matrix metallopeptidase 13; MOD, mean optical density; Col2a1, collagen type II alpha 1; Mmp3, matrix metallopeptidase 3; Adamts5, ADAM metallopeptidase with thrombospondin type 1 motif 5; RT-qPCR, real-time quantitative polymerase chain reaction; PW, postnatal week.

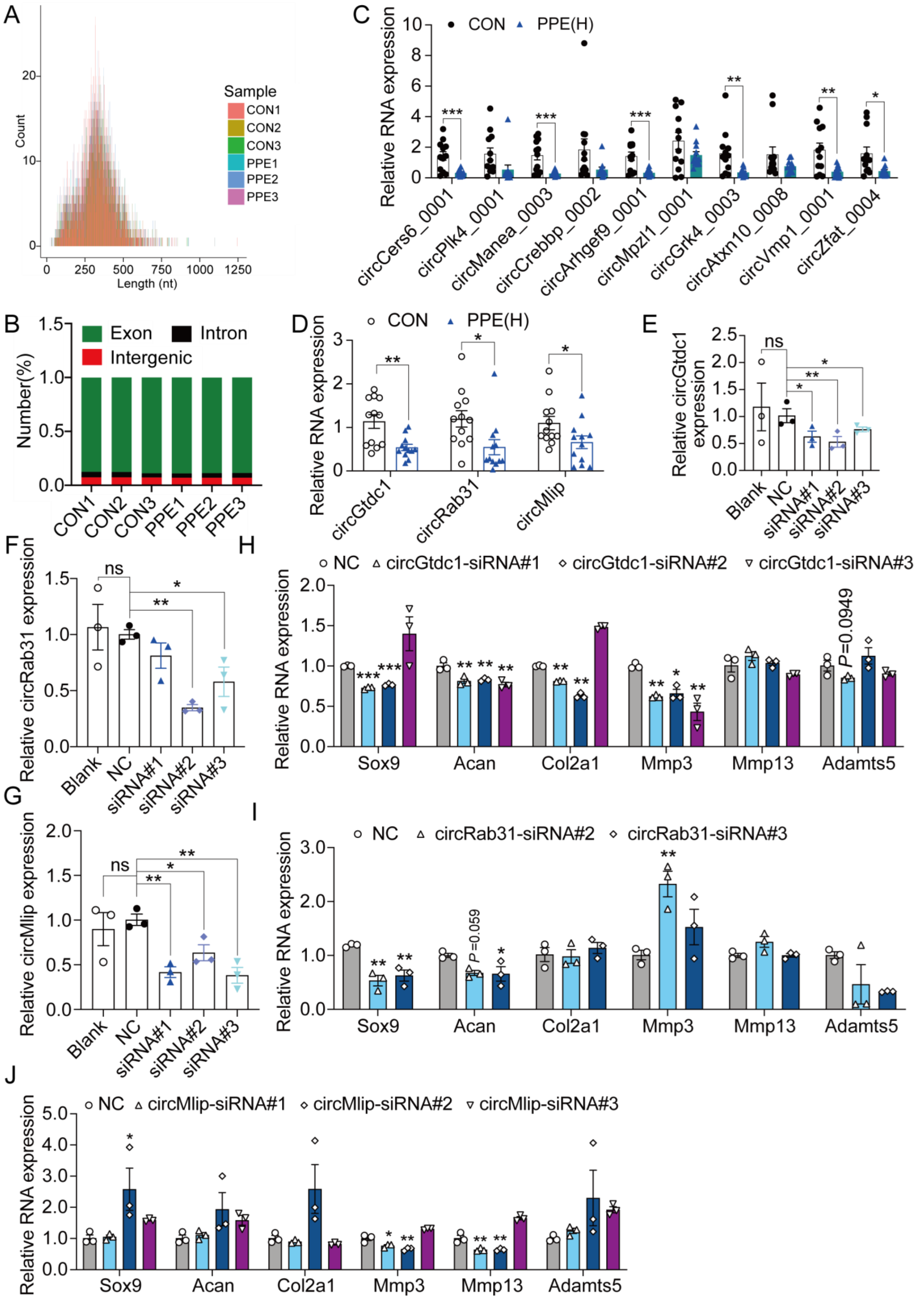

Fig. S2. Effects of PPE on circRNomics in cartilage. (A) The length distribution of circRNA by sequencing; (B) The classification

ration of circRNA obtained by sequencing in cartilage; RT-qPCR detected the circRNA expression in cartilage at GD20 (C) and PW28 (D), n=12; (E-G) RT-qPCR detected the circGtdc1, circRab31, and circMlip expression in fetal rat chondrocytes treated by the respective specific siRNAs and the relative expression of the NC group was designated as 1, n=3; RT-qPCR detected the mRNA expression levels of Sox9, Acan, Col2a1, Mmp3, Mmp13 and Adamts5 in chondrocytes treated by siRNAs of circGtdc1 (H), circRab31 (I) and circMlip (J), n=3; Values are expressed as the means ± S.E.M. $^{*}P < 0.05$, $^{**}P < 0.01$, $^{***}P < 0.001$ *vs.* corresponding control. PPE, prenatal prednisone expression; CON, control; GD, gestational day; PW, postnatal week; NC, negative control; circGtdc1, circular RNA Gtdc1; circRab31, circular RNA Rab31; circMlip, circular RNA Mlip; Sox9, SRY (sex-determining region Y)-box9; Col2a1, collagen type II alpha 1; Acan, aggrecan; Mmp3, matrix metallopeptidase 3; Mmp13, matrix metallopeptidase 13; Adamts5, ADAM metallopeptidase with thrombospondin type 1 motif 5; RT-qPCR, real-time quantitative polymerase chain reaction.

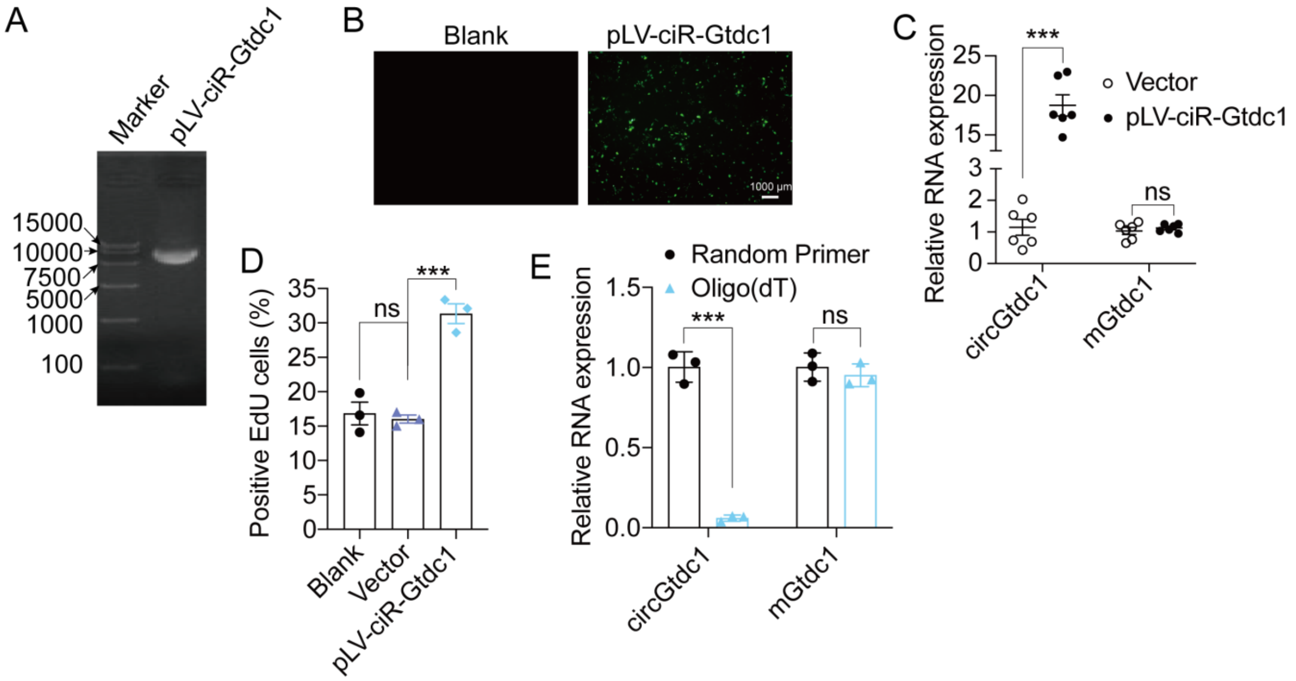

Fig. S3. Overexpression of circGtdc1 and its effects on chondrocytes. (A) Agarose gel electrophoresis verified the plasmid of circGtdc1; (B) Immunofluorescence confirmed the expression of pLV-ciR-Gtdc1 in chondrocytes; (C) The circGtdc1 and mGtdc1 expression in fetal rat chondrocytes treated by circGtdc1 overexpression plasmid (pLV-ciR-Gtdc1), n=3×2; (D) The chondrocyte number of positive EdU staining, n=3; (E) The circGtdc1 and mGtdc1 expression in cDNA amplified by Oligo(dT) and random primers, n=3.Values are expressed as the means ± S.E.M. $^{**}P < 0.01$, $^{***}P < 0.001$ *vs.* corresponding control. circGtdc1, circular RNA Gtdc1; mGtdc1, mRNA of glycosyltransferase like domain containing 1; EdU, 5-ethynyl-2'-deoxyuridine; cDNA, complementary DNA.

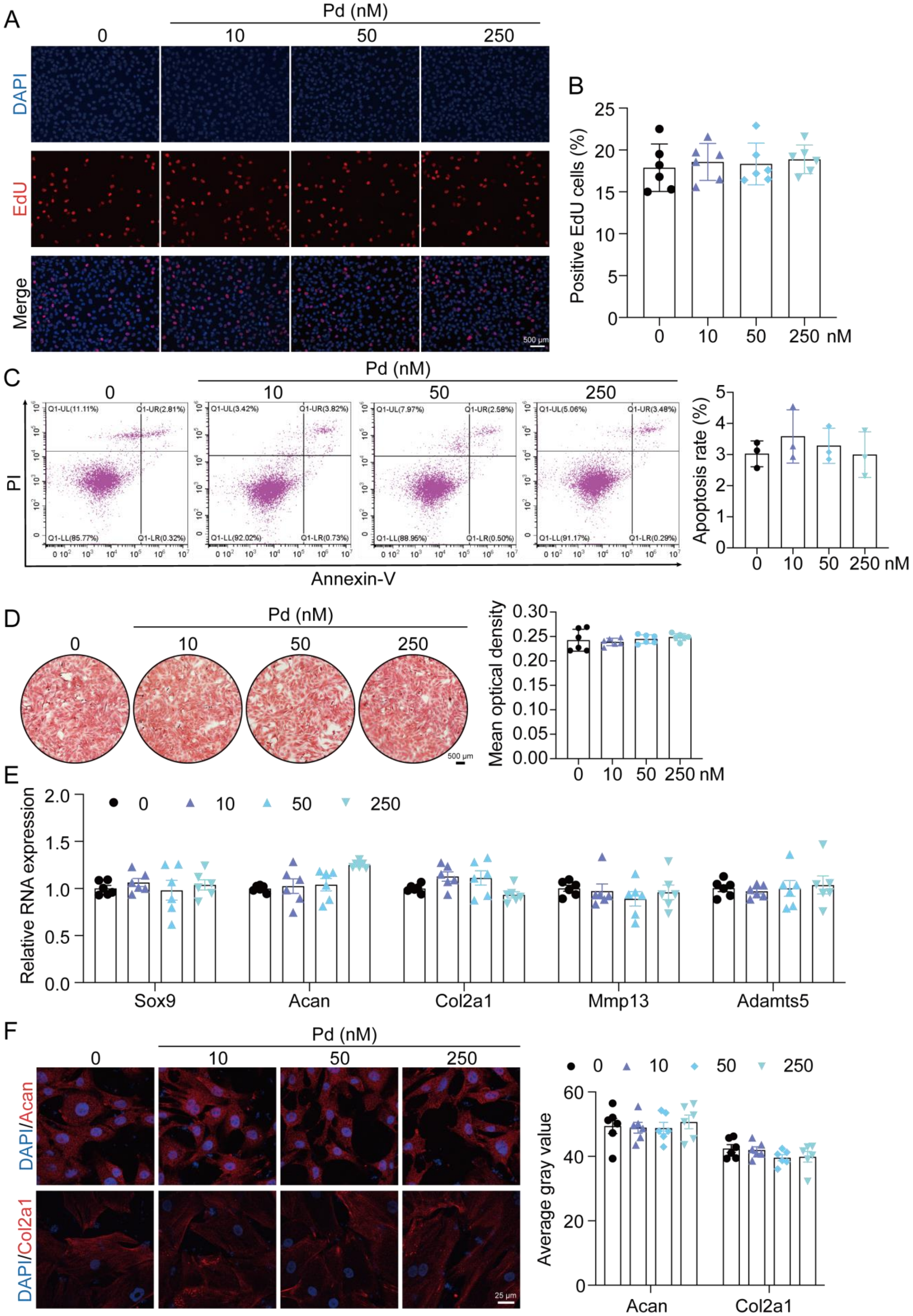
A
Pd (nM)
0
10
50
250
DAPI
EdU
Merge
500 μm
B
Positive EdU cells (%)
0 10 50 250 nM
C
Pd (nM)
PI
Annexin-V
Q1-UL(11.11%)
Q1-UR(2.81%)
Q1-LL(85.77%)
Q1-LR(0.32%)
Q1-UL(3.42%)
Q1-UR(3.82%)
Q1-LL(92.02%)
Q1-LR(0.73%)
Q1-UL(7.97%)
Q1-UR(2.58%)
Q1-LL(88.95%)
Q1-LR(0.50%)
Q1-UL(5.06%)
Q1-UR(3.48%)
Q1-LL(91.17%)
Q1-LR(0.29%)
Apoptosis rate (%)
D
Pd (nM)
500 μm
Mean optical density
E
Relative RNA expression
Sox9
Acan
Col2a1
Mmp13
Adamts5
F
Pd (nM)
DAPI/Acan
DAPI/Col2a1
25 μm
Average gray value
Acan
Col2a1

Fig. S4. Influences of prednisone in proliferation, apoptosis and matrix synthesis of fetal rat chondrocytes. (A) EdU was used to assay the proliferation ability of fetal rat primary chondrocytes treated as indicated; (B) The chondrocyte number of positive EdU staining, n=6; (C) Flow cytometry was used to assay apoptosis of chondrocytes, n=3; (D) Safranin O staining was used to assay matrix contents of chondrocytes induced by prednisone, n=6; (E) RT-qPCR was applied to confirm the mRNA expression of Sox9, Acan, Col2a1, Mmp13 and Adamts5 in chondrocytes treated as indicated, n=6; (F) Immunofluorescence was used to confirm the protein expression of Acan and Col2a1, n=6. Values are expressed as the means ± S.E.M. $^{*}P < 0.05$, $^{**}P < 0.01$, $^{***}P < 0.001$ *vs.* corresponding control. EdU, 5-ethynyl-2'-deoxyuridine; Pd, prednisone; Sox9, SRY (sex-determining region Y)-box9; Col2a1, collagen type II alpha 1; Acan, aggrecan; Mmp13, matrix metallopeptidase 13; Adamts5, ADAM metallopeptidase with thrombospondin type 1 motif 5; RT-qPCR, real-time quantitative polymerase chain reaction.

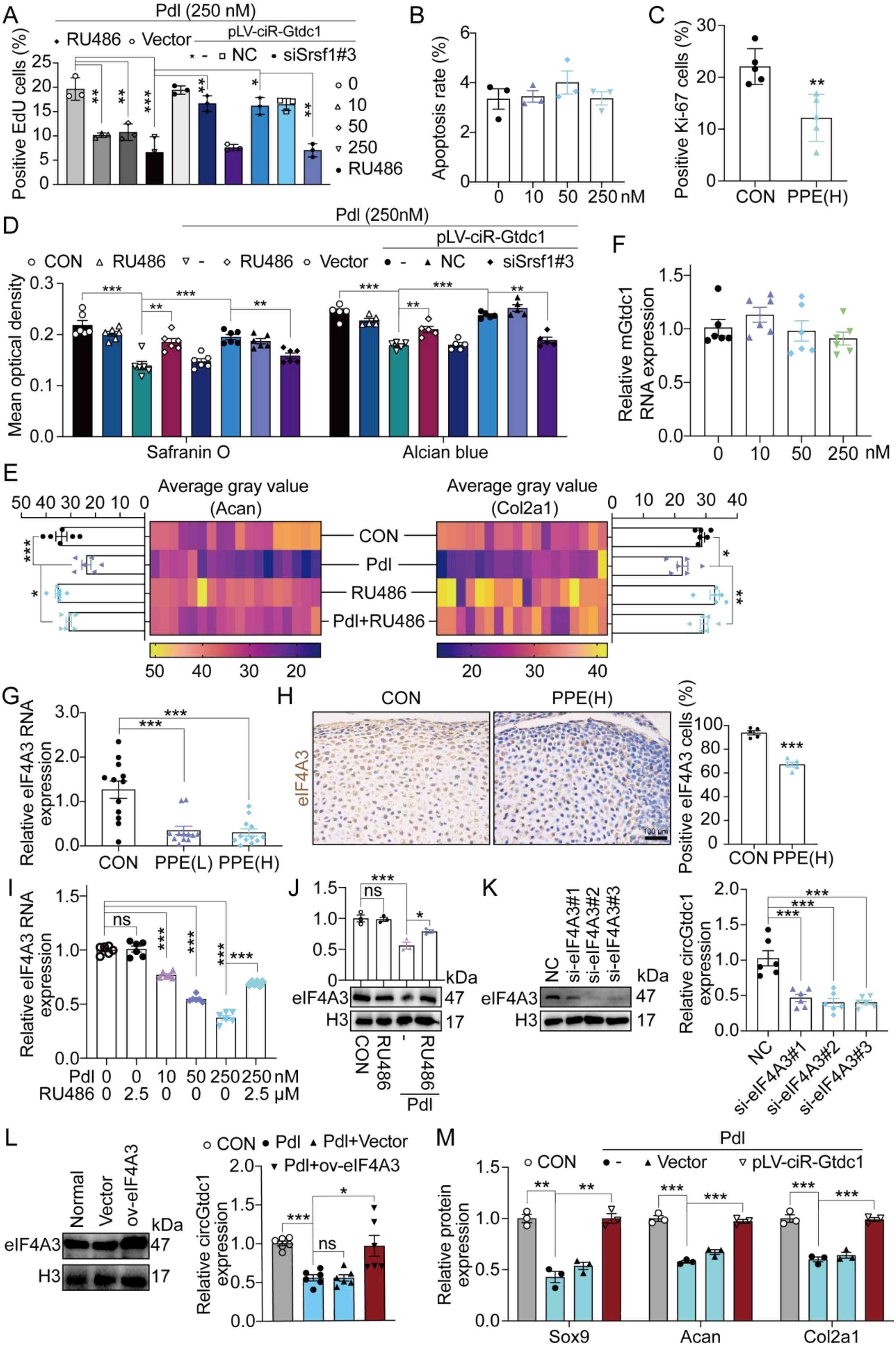
A
Pdl (250 nM)
RU486 Vector pLV-ciR-Gtdc1
- NC siSrsf1#3
Positive EdU cells (%)
0 10 50 250 RU486
B
Apoptosis rate (%)
0 10 50 250 nM
C
Positive Ki-67 cells (%)
CON PPE(H)
D
Pdl (250nM)
pLV-ciR-Gtdc1
CON RU486 - RU486 Vector - NC siSrsf1#3
Mean optical density
Safranin O
Alcian blue
E
Average gray value (Acan)
Average gray value (Col2a1)
CON
Pdl
RU486
Pdl+RU486
F
Relative mGtdc1 RNA expression
0 10 50 250 nM
G
Relative eIF4A3 RNA expression
CON PPE(L) PPE(H)
H
CON PPE(H)
eIF4A3
Positive eIF4A3 cells (%)
CON PPE(H)
I
Relative eIF4A3 RNA expression
Pdl 0 0 10 50 250 250 nM
RU486 0 2.5 0 0 0 2.5 μM
J
kDa
eIF4A3 47
H3 17
CON RU486 - RU486
Pdl
K
NC si-eIF4A3#1 si-eIF4A3#2 si-eIF4A3#3
kDa
eIF4A3 47
H3 17
Relative circGtdc1 expression
L
Normal Vector ov-eIF4A3
kDa
eIF4A3 47
H3 17
CON Pdl Pdl+Vector Pdl+ov-eIF4A3
Relative circGtdc1 expression
M
Pdl
CON - Vector pLV-ciR-Gtdc1
Relative protein expression
Sox9 Acan Col2a1

Fig. S5. Influences of prednisolone in proliferation and matrix synthesis of chondrocytes. (A) The chondrocyte number of positive EdU staining, n=3; (B) The apoptosis rate analysis by Flow cytometry in fetal rat chondrocytes treated with prednisolone, n=3; (C) The chondrocyte number of positive Ki-67 assayed by immunohistochemical staining, n=5; (D) The quantification (MOD) of Safranin O (n=6) and Alcian blue (n=5) staining in prednisolone-induced chondrocytes treated by pLV-ciR-Gtdc1 combined with or without siRNA of Srsf1; (E) The statistical analysis of Acan and Col2a1 expression by immunofluorescence, n=6; (F) The mGtdc1 expression in chondrocytes treated by different concentrations of prednisolone for 48 h, n=6; (G) RT-qPCR was applied to confirm the mRNA expression of eIF4A3 in cartilage of fetal rats, n=12; (H) Immunohistochemistry was applied to assay the protein expression of eIF4A3 in cartilage on GD20 and MOD was sued for quantification, n=5; (I-J) RT-qPCR and western blotting was used to assay the mRNA (n=6) and protein expression of eIF4A3 (n=3) in primary chondrocytes treated as indicated; (K-L) RT-qPCR was applied to confirm the eIF4A3 mRNA expression in chondrocytes treated with eIF4A3 siRNAs (si-eIF4A3) or overexpression plasmids (ov-eIF4A3), n=6; (M) The statistical analysis of Sox9, Acan and Col2a1 assayed by western blotting, n=3. Values are expressed as the means ± S.E.M. $^{*}P < 0.05$, $^{**}P < 0.01$, $^{***}P < 0.001$ *vs.* corresponding control. EdU, 5-ethynyl-2'-deoxyuridine; Pdl, prednisolone; NC, negative control; Gtdc1, glycosyltransferase like domain containing 1; Srsf1, serine and arginine rich splicing factor 1; Ki-67, marker of proliferation Ki-67; CON, control; PPE, prenatal prednisone expression; NC, negative control; mGtdc1, mRNA of glycosyltransferase like domain containing 1; Acan, aggrecan; Col2a1, collagen type II alpha 1; eIF4A3, eukaryotic translation initiation factor 4A3; Sox9, SRY (sex-determining region Y)-box9.

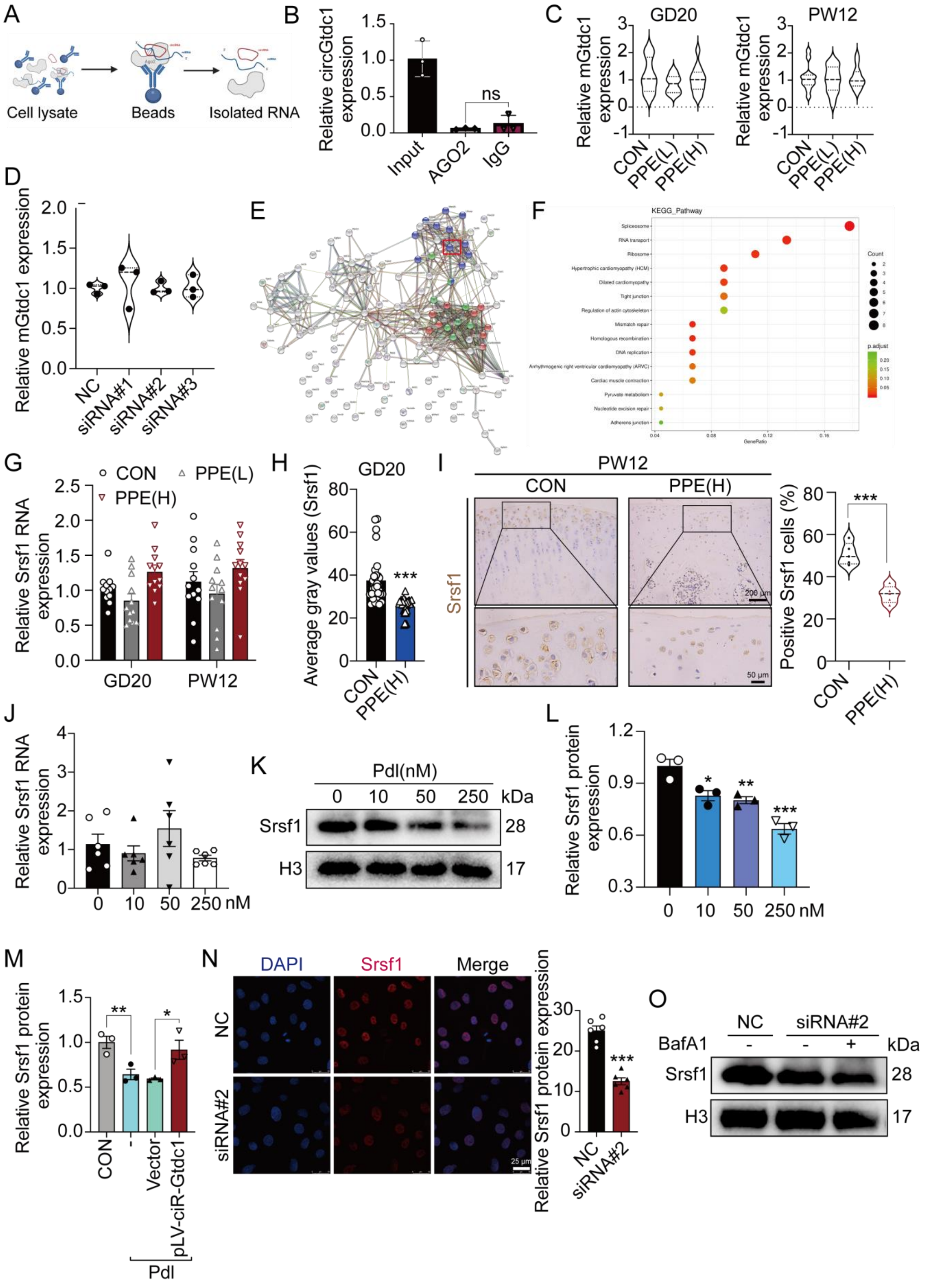

Fig. S6. Influences of circGtdc1 on Srsf1 protein stability. (A) Schemes of RNA immunoprecipitation assay; (B) The binding

between circGtdc1 and AGO2 assayed by RNA immunoprecipitation assay, n=3; (C) The mGtdc1 expression in rat cartilage induced by PPE, n=12; (D) mGtdc1 expression in chondrocytes treated by siRNAs of circGtdc1, n=3; (E) Protein interaction analysis of mass spectrometry results by an online tool STRING; (F) KEGG analysis of mass spectrometry after RNA pulldown by circGtdc1 in chondrocytes; (G) The mRNA expression levels of Srsf1 in rat cartilage treated as indicated, n=12; (H) The statistical analysis of Srsf1 protein levels in cartilage on GD20 assayed by immunofluorescence, n=5×6; (I) The protein expression levels of Srsf1 in rat cartilage at PW12 was assayed by immunohistochemical staining, n=5; (J)The mRNA expression levels of Srsf1 in chondrocytes treated with different concentrations of prednisolone (0, 10, 50, and 250 nM), n=6; (K-L) The protein expression levels of Srsf1 and statistical analysis by western blotting in chondrocytes treated by different concentrations of prednisolone, n=3; (M) The statistical analysis of Srsf1 protein levels in chondrocytes assayed by western blotting, n=3; (N) Immunofluorescence was used to determine protein expression of Srsf1, n=6; (O) The protein expression levels of Srsf1 in chondrocytes treated by circGtdc1 siRNA and BafA1 (10 μM for 2 h). Values are expressed as the means ± S.E.M. $^{*}P < 0.05$, $^{**}P < 0.01$, $^{***}P < 0.001$ *vs.* corresponding control. circGtdc1, circular RNA Gtdc1; Srsf1, serine and arginine rich splicing factor 1; AGO2, argonaute 2; IgG, immunoglobulin G; CON, control; PPE, prenatal prednisone expression; mGtdc1, mRNA of glycosyltransferase like domain containing 1; GD, gestational day; PW, postnatal week; Pdl, prednisolone; NC, negative control; BafA1, bafilomycin A1.

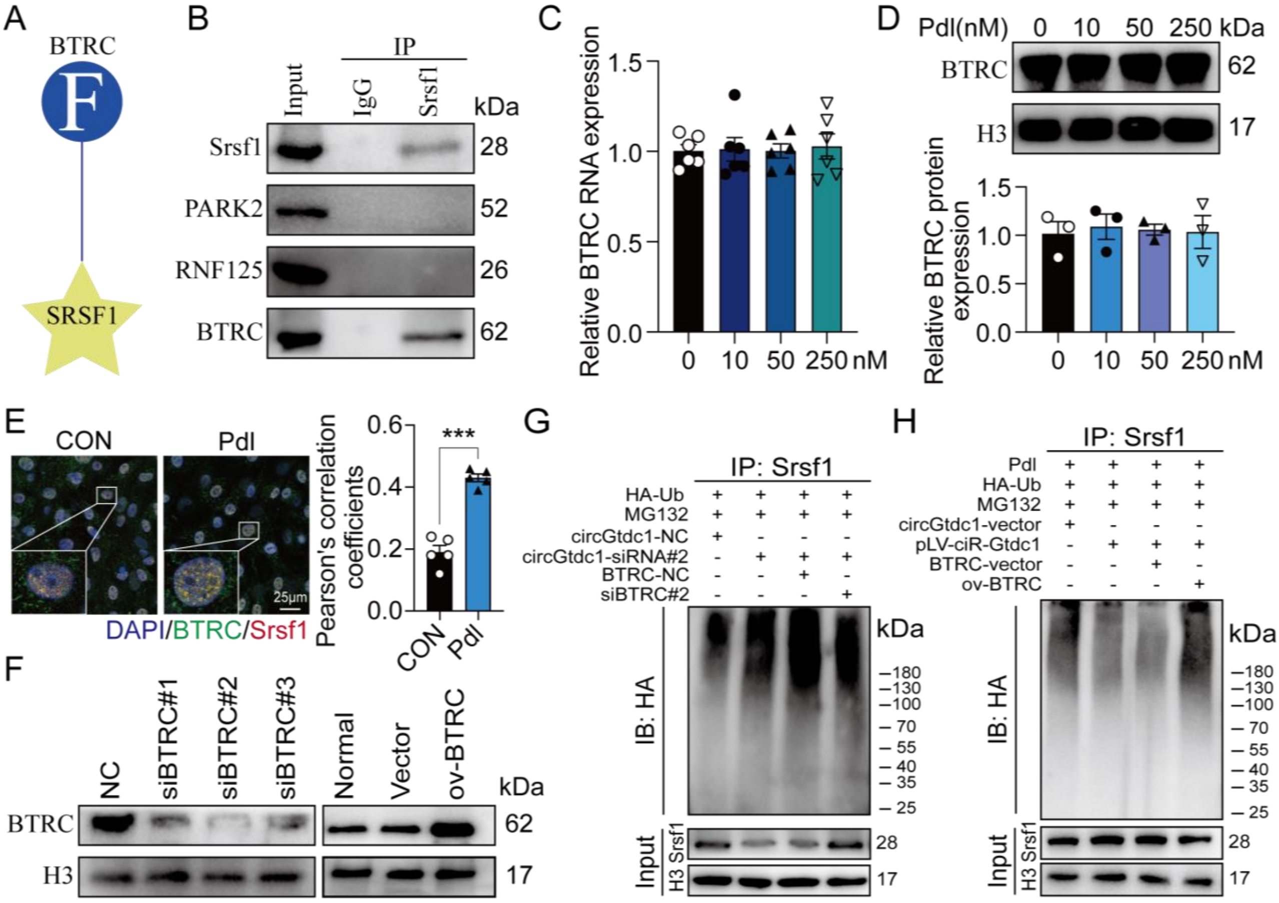

Fig. S7. Effects of BTRC on Srsf1 ubiquitination caused by low circGtdc1. (A) E3 ligases were predicted by UbiBrowser database; (B) The binding between PARK2, RNF123, BTRC and Srsf1 protein was assayed by Co-IP subsequent western blotting; (C) RT-qPCR was used to detected the mRNA expression of BTRC, n=6; (D) Western blotting was performed to detect BTRC protein levels in chondrocytes treated as indicated, n=3; (E) Immunofluorescence was performed to assay the colocalization between Srsf1 and BTRC in chondrocytes treated with prednisolone (250 nM) and the Pearson's correlation coefficients were statistically analyzed, n=5; (F) Western blotting was performed to detect BTRC protein levels in chondrocytes treated by BTRC knockdown or overexpression with corresponding siRNAs and plasmids; (G) The ubiquitination of Srsf1 protein in fetal primary chondrocytes treated with circGtdc1 and/or BTRC siRNAs; (H) Western blotting and IP showing the effects of circGtdc1 and/or BTRC overexpression on the ubiquitination of Srsf1 protein in fetal rat primary chondrocytes induced by prednisolone (250 μM). Values are expressed as the means ± S.E.M. $^{*}P$ < 0.05, $^{**}P < 0.01$, $^{***}P < 0.001$ *vs.* corresponding control. BTRC, beta-transducin repeat containing E3 ubiquitin protein; Srsf1, serine

and arginine rich splicing factor 1; IP: immunoprecipitation; IgG, immunoglobulin G; PARK2, parkin RBR E3 ubiquitin protein ligase; RNF125, ring finger protein 125; Pdl, prednisolone; CON, control; Ub, ubiquitin; IB: immunoblot; HA, hyaluronic acid; NC, negative control RT-qPCR, real-time quantitative polymerase chain reaction.

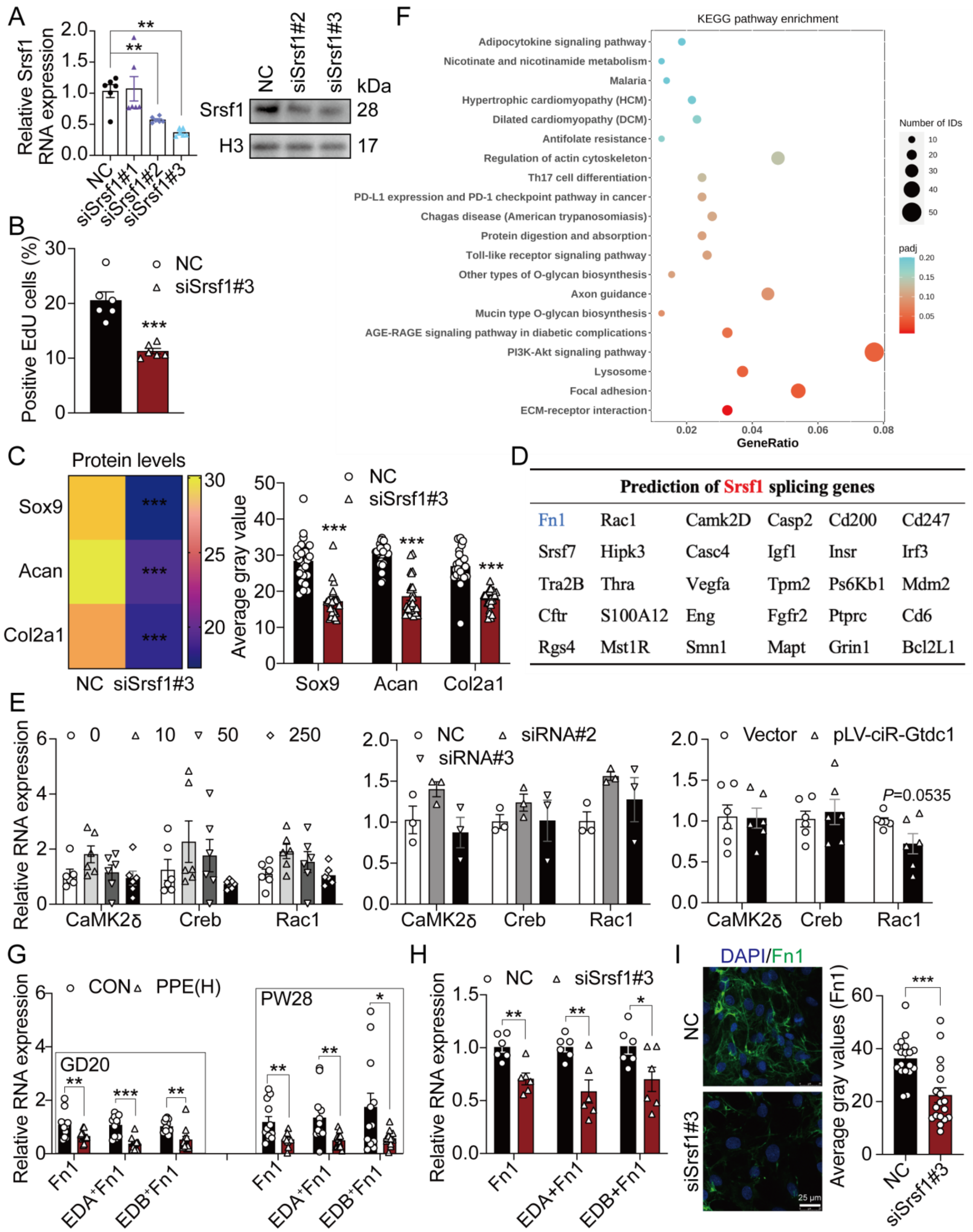

Fig. S8. Influences of Srsf1 in alternative splicing of Fn1 in chondrocytes. (A) The mRNA (n=6) and protein expression of Srsf1 in

chondrocytes treated by Srsf1 siRNA; (B) The chondrocyte number of positive EdU staining, n=6; (C) The statistical analysis of Sox9, Acan and Col2a1 assayed by immunofluorescence, n=6×4; (D) Srsf1 splicing genes predicted by miasDB database; (E) The mRNA expression of CaMK2δ, Creb, and Rac1 in chondrocytes treated by prednisolone, Srsf1 siRNA or circGtdc1 overexpression plasmid (pLV-ciR-Gtdc1), n=3 or 6; (F) KEGG analysis of RNA sequencing after Srsf1 siRNA treatment in chondrocytes; (G) RT-qPCR was used to determine the RNA level of Fn1, EDA^{+}Fn1 and EDB^{+}Fn1 in rat cartilage induced by PPE, n=12; (H) The levels of Fn1, EDA^{+}Fn1 and EDB^{+}Fn1 in chondrocytes treated by Srsf1 siRNA, n=6; (I) Immunofluorescence was used to assay the protein level of Fn1 in chondrocytes treated by Srsf1 siRNA, n=3×6. Values are expressed as the means ± S.E.M. $^{*}P < 0.05$, $^{**}P < 0.01$, $^{***}P < 0.001$ *vs.* corresponding control. Srsf1, serine and arginine rich splicing factor 1; Fn1, fibronectin 1; NC, negative control; EdU, 5-ethynyl-2'-deoxyuridine; Sox9, SRY (sex-determining region Y)-box9; Col2a1, collagen type II alpha 1; Acan, aggrecan; CaMK2δ, Calcium/Calmodulin-dependent protein kinase (CaM Kinase) II delta; Creb, CAMP responsive element binding protein 1; Rac1, Rac family small GTPase 1; CON, control; PPE, prenatal prednisone expression; GD, gestational day; PW, postnatal week; RT-qPCR, real-time quantitative polymerase chain reaction.

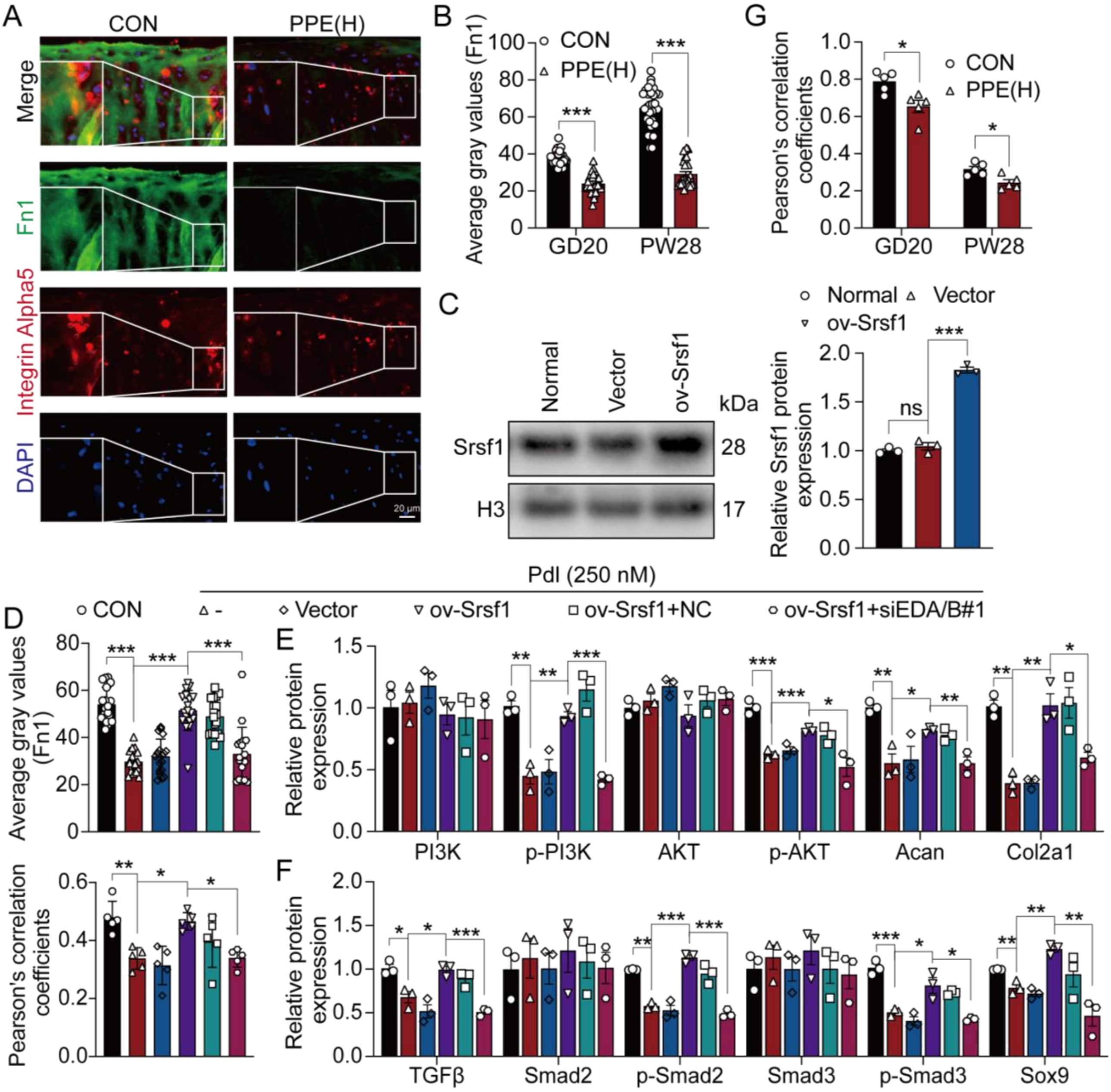

Fig. S9. Effects of Srsf1 on Fn1 and ITGα5 in PPE induced rat cartilage and PI3K/AKT and TGFβ signaling in chondrocytes

treated by prednisolone. (A) Immunofluorescence was performed to assay the colocalization between Fn1 and ITGα5 in rat cartilage tissue at PW28, n=5; (B) The statistical analysis of Fn1 protein level in PPE-induced rat cartilage tissue on GD20 and at PW28, n=5×6; (C) The protein level of Srsf1 was assayed by western blotting in Srsf1 overexpressed chondrocytes, n=3; (D) The statistical analysis of Fn1 protein level (n=5×4) and colocalization between Fn1 and integrin alpha 5 in fetal primary chondrocytes with Srsf1 overexpression induced by prednisolone (250 nM) (n=5); (E-F) The statistical analysis of PI3K, p-PI3K, AKT, p-AKT, Acan, Col2a1, TGFβ, Smad2, p-Smad2, Smad3, p-Smad3 and Sox9 protein level in fetal primary chondrocytes treated with Srsf1 overexpression plasmid and/or Fn1 siRNA, n=3; (G) The statistical analysis of colocalization between Fn1 and integrin alpha 5 in PPE-induced rat cartilage tissue on GD20 and at PW28, n=5.Values are expressed as the means ± S.E.M. $^{*}P < 0.05$, $^{**}P < 0.01$, $^{***}P < 0.001$ *vs.* corresponding control. Srsf1, serine and arginine rich splicing factor 1; ITGα5, integrin receptor α5; PI3K, phosphoinositide 3-kinase; AKT, AKT serine/threonine kinase; TGFβ, transforming growth factor beta; Fn1, fibronectin 1; CON, control; PPE, prenatal prednisone expression; GD, gestational day; PW, postnatal week; Pdl, prednisolone; p-PI3K, phospho-phosphoinositide 3-kinase; p-AKT, phospho-AKT; Smad2, SMAD family member 2; p-Smad2, phospho-SMAD family member 2; Smad3, SMAD family member 3; p-Smad3, phospho-SMAD family member 3; Acan, aggrecan; Col2a1, collagen type II alpha 1; Sox9, SRY (sex-determining region Y)-box9.

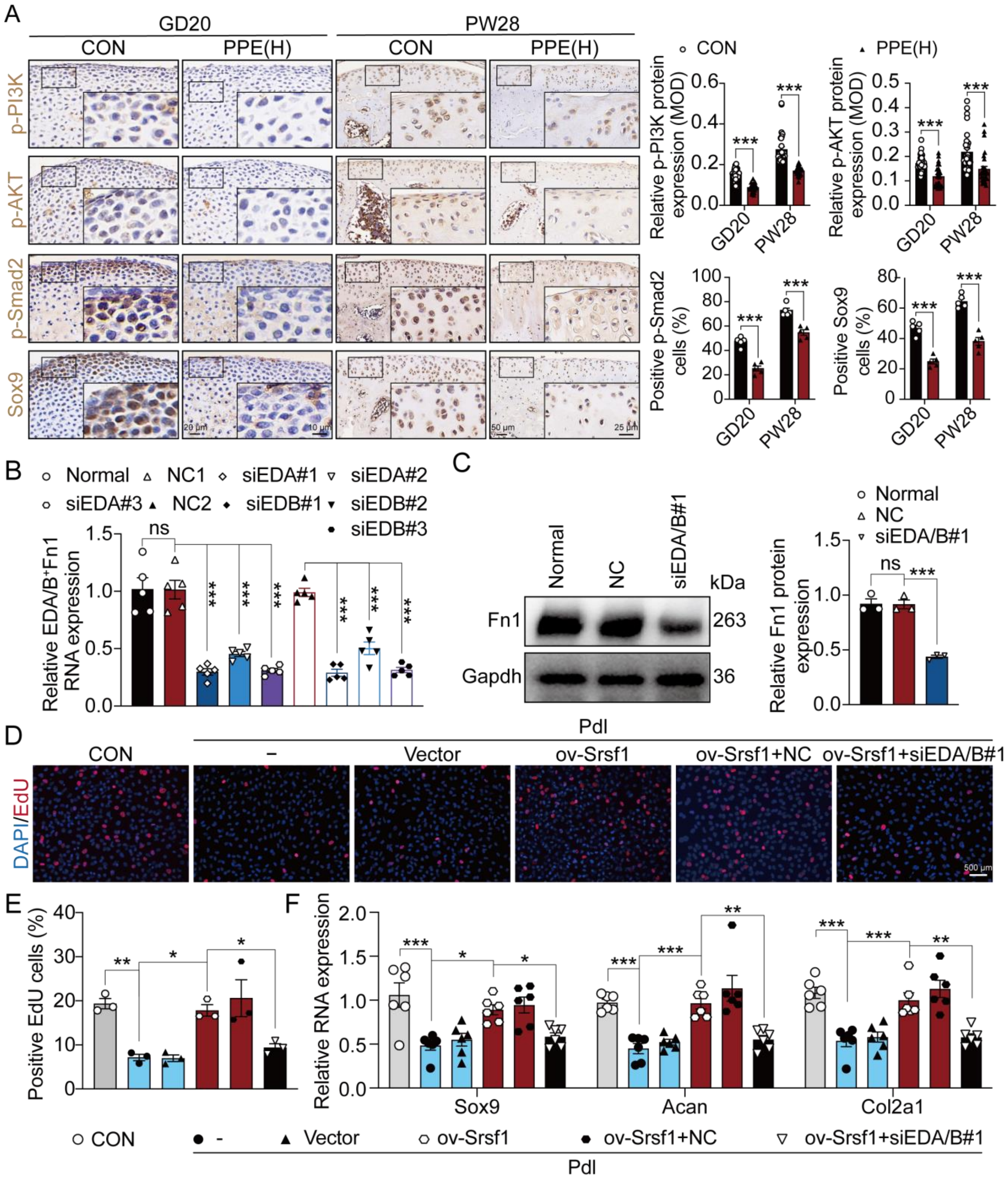

Fig. S10. Effects on PPE on activation of PI3K/AKT and TGFβ pathway in cartilage and Srsf1-Fn1 signaling on proliferation and extracellular matrix synthesis of chondrocytes induced by prednisolone. (A) Immunohistochemical staining and statistical analysis of p-PI3K, p-AKT, p-Smad2, and Sox9 protein in cartilage treated on GD20 and PW28, n=5 or 5×6; (B) The RNA expression of EDA/B$^+$Fn1 was determine by RT-qPCR in chondrocytes treated by EDA/B$^+$Fn1 siRNA, n=5; (C) The protein level of Fn1 was assayed by western blotting in EDA/B$^+$Fn1 knockdown chondrocytes, n=3; (D) EdU was used to assay the proliferation ability of fetal rat primary chondrocytes treated as indicated; (E) Statistical analysis of EdU staining, n=3; (F) RT-qPCR was used to confirmed the mRNA expression of Sox9, Acan, and Col2a1, n=6. Values are expressed as the means ± S.E.M. $^*P < 0.05$, $^{**}P < 0.01$, $^{***}P < 0.001$ *vs.* corresponding control. PI3K, phosphoinositide 3-kinase; AKT, AKT serine/threonine kinase; TGFβ, transforming growth factor beta; Srsf1, serine and arginine rich splicing factor 1; Fn1, fibronectin 1; CON, control; PPE, prenatal prednisone expression; GD, gestational day; PW, postnatal week; p-PI3K, phospho-phosphoinositide 3-kinase; p-AKT, phospho-AKT; p-Smad2, phospho-SMAD family

member 2; Sox9, SRY (sex-determining region Y)-box9; MOD, mean optical density; NC, negative control; Pdl, prednisolone; Gapdh, glyceraldehyde 3-phosphate dehydrogenase; EdU, 5-ethynyl-2'-deoxyuridine; Srsf1, serine and arginine rich splicing factor 1; Col2a1, collagen type II alpha 1; Acan, aggrecan.

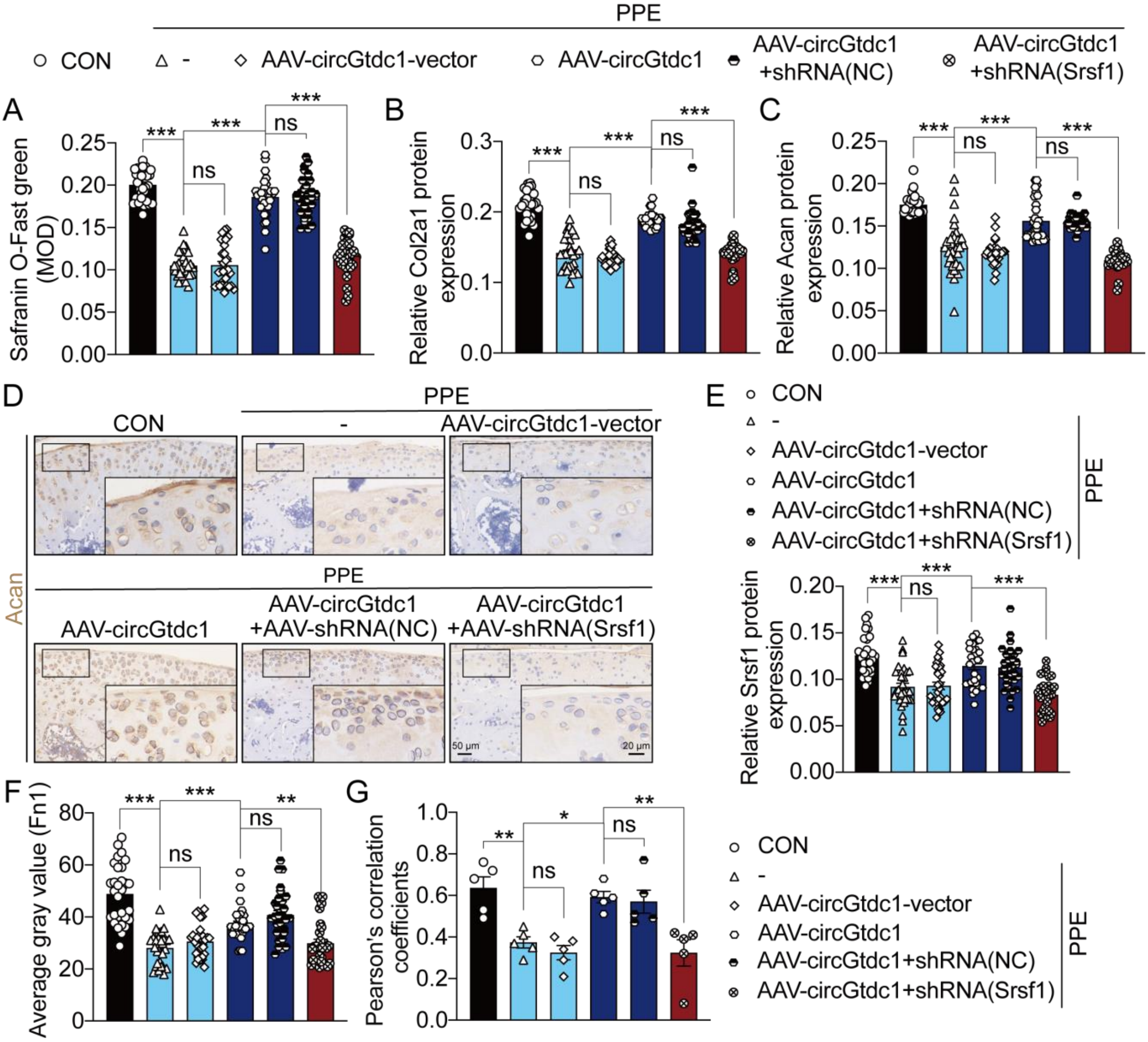

Fig. S11. Influences of circGtdc1 overexpression combined with or without Srsf1 knockdown in rat cartilage induced by PPE. (A) The statistical analysis of Safranin O-Fast Green staining of cartilage in PPE-induced rats treated with AAV-circGtdc1 and/or AAV-shRNA(Srsf1) during PW8-12, n=5×6; (B-C) The statistical analysis of Col2a1 and Acan protein levels assayed by immunohistochemical staining, n=5×6; (D) Immunohistochemical staining was applied to assay the protein level of Acan in cartilage treated as indicated, n=5; (E) The statistical analysis of Srsf1 protein level in cartilage treated as indicated, n=5×6; (F-G) The statistical analysis of Fn1 protein level (n=5×6) and colocalization (n=5) between Fn1 and integrin alpha 5 in cartilage of PPE-induced rats treated with AAV-circGtdc1 and/or AAV-shRNA(Srsf1) during PW8-12. Values are expressed as the means ± S.E.M. $^{*}P < 0.05$, $^{**}P < 0.01$, $^{***}P < 0.001$ *vs.* corresponding control. circGtdc1, circular RNA Gtdc1; Srsf1, serine and arginine rich splicing factor 1; PPE, prenatal prednisone expression; MOD, mean optical density; Col2a1, collagen type II alpha 1; Acan, aggrecan; CON, control; AAV, adeno-associated virus; NC, negative control; Fn1, fibronectin 1.

References:

[1] J. Martel-Pelletier, A. J. Barr, F. M. Cicuttini, P. G. Conaghan, C. Cooper, M. B. Goldring, S. R. Goldring, G. Jones, A. J. Teichtahl, J. P. Pelletier, *Nat Rev Dis Primers* **2016**, *2*, 16072, https://doi.org/10.1038/nrdp.2016.72.

[2] Q. Li, Y. Wen, L. Wang, B. Chen, J. Chen, H. Wang, L. Chen, *Exp Mol Med* **2021**, *53* (11), 1735, https://doi.org/10.1038/s12276-021-00697-6.

[3] Y. Xu, G. Mao, D. Long, Z. Deng, R. Xin, Z. Zhang, T. Xue, W. Liao, J. Xu, Y. Kang, *Exp Mol Med* **2022**, *54* (10), 1727, https://doi.org/10.1038/s12276-022-00865-2.

[4] a) R. L. Taylor, S. K. Grebe, R. J. Singh, *Clin Chem* **2004**, *50* (12), 2345, https://doi.org/10.1373/clinchem.2004.033605; b) M. K. Auer, A. Krumbholz, M. Bidlingmaier, D. Thieme, N. Reisch, *Neuroendocrinology* **2020**, *110* (11-12), 938, https://doi.org/10.1159/000504672.

[5] Y. J. Liao, P. C. Tang, L. R. Chen, J. R. Yang, *J Histotechnol* **2020**, *43* (4), 204, https://doi.org/10.1080/01478885.2020.1756081.

[6] L. Liu, B. Li, Q. Li, H. Han, S. Zhou, Z. Wu, H. Gao, J. Zhu, H. Gu, L. Chen, H. Wang, *J Adv Res* **2022**, https://doi.org/10.1016/j.jare.2022.08.002.

[7] K. P. Pritzker, S. Gay, S. A. Jimenez, K. Ostergaard, J. P. Pelletier, P. A. Revell, D. Salter, W. B. van den Berg, *Osteoarthr Cartilage* **2006**, *14* (1), 13, https://doi.org/10.1016/j.joca.2005.07.014.

[8] a) T. D. Schmittgen, K. J. Livak, *Nat Protoc* **2008**, *3* (6), 1101, https://doi.org/10.1038/nprot.2008.73; b) K. J. Livak, T. D. Schmittgen, *Methods* **2001**, *25* (4), 402, https://doi.org/10.1006/meth.2001.1262.

[9] S. Memczak, M. Jens, A. Elefsinioti, F. Torti, J. Krueger, A. Rybak, L. Maier, S. D. Mackowiak, L. H. Gregersen, M. Munschauer, A. Loewer, U. Ziebold, M. Landthaler, C. Kocks, F. le Noble, N. Rajewsky, *Nature* **2013**, *495* (7441), 333, https://doi.org/10.1038/nature11928.

[10] Y. Gao, J. Wang, F. Zhao, *Genome Biol* **2015**, *16* (1), 4, https://doi.org/10.1186/s13059-014-0571-3.

[11] K. Orzechowska, G. Kopij, L. Paukszto, K. Dobrzyn, M. Kiezun, J. Jastrzebski, T. Kaminski, N. Smolinska, *Biol Reprod* **2022**, *107* (2), 557, https://doi.org/10.1093/biolre/ioac063.

[12] L. Qing-Xian, W. Lin-Long, W. Yi-Zhong, L. Liang, H. Hui, C. Liao-Bin, W. Hui, *Pharmacol Res* **2020**, *151*, 104555, https://doi.org/10.1016/j.phrs.2019.104555.

[13] a) Y. Liao, J. T. Long, C. J. R. Gallo, A. J. Mirando, M. J. Hilton, *Methods Mol Biol* **2021**, *2230*, 415, https://doi.org/10.1007/978-1-0716-1028-2_25; b) A. Ramser, E. Greene, N. Rath, S. Dridi, *Poult Sci* **2023**, *102* (1), 102254, https://doi.org/10.1016/j.psj.2022.102254; c) M. Gosset, F. Berenbaum, S. Thirion, C. Jacques, *Nat Protoc* **2008**, *3* (8), 1253, https://doi.org/10.1038/nprot.2008.95.

[14] Z. Yu, Y. Lv, C. Su, W. Lu, R. Zhang, J. Li, B. Guo, H. Yan, D. Liu, Z. Yang, H. Mi, L. Mo, Y. Guo, W. Feng, H. Xu, W. Peng, J. Cheng, A. Nan, Z. Mo, *Cancer Res* **2023**, *83* (5), 700, https://doi.org/10.1158/0008-5472.Can-22-2224.

[15] Q. Zhao, J. Liu, H. Deng, R. Ma, J. Y. Liao, H. Liang, J. Hu, J. Li, Z. Guo, J. Cai, X. Xu, Z. Gao, S. Su, *Cell*

2020, *183* (1), 76, https://doi.org/10.1016/j.cell.2020.08.009.

[16] L. Liu, H. Geng, C. Mei, L. Chen, *Oxid Med Cell Longev* **2021**, *2021*, 6661534, https://doi.org/10.1155/2021/6661534.
